# Supplementary material for: Neural circuitry of social learning in Drosophila requires multiple inputs to facilitate inter-species communication
Source: Commun Biol. 2019 Aug 13;2:309. doi: 10.1038/s42003-019-0557-5 (PMC6692349; doi:10.1038/s42003-019-0557-5)
Supplement: Supplementary file 1 — Supplementary Material [file 42003_2019_557_MOESM1_ESM.pdf]

SUPPLEMENTARY FIGURES

Supplementary Figure 1. Wild-type *D. melanogaster* can be trained by *D. ananassae* at either 22°C or 30°C.

Dialect training is performed at either the permissive (22°C) or restrictive (30°C) temperature, while the wasp exposure and social learning period is performed exclusively at the permissive temperature. Percentage of eggs laid by exposed flies normalized to eggs laid by unexposed flies is shown. *Canton S* untrained and trained states by *D. ananassae* at either 30°C (A) or 22°C (B) show wild-type dialect acquisition. Blue lines indicate permissive temperature, while red lines indicate restrictive temperature. Trained and untrained states are labeled. Plots are standard Tukey Box Plots, where the bounds of the shaded box represent the first and third quartile; whiskers indicate the minimum and maximum data point within 1.5 times the interquartile range. Mean is denoted with an 'X.' Data used to generate the plots have been superimposed on the graph as open circles (n = 12 biologically independent experiments) (*$p < 0.05$, a→ $p < 0.05$ comparing trained and untrained students within a given temperature when both restrictive and permissive temperatures are tested).

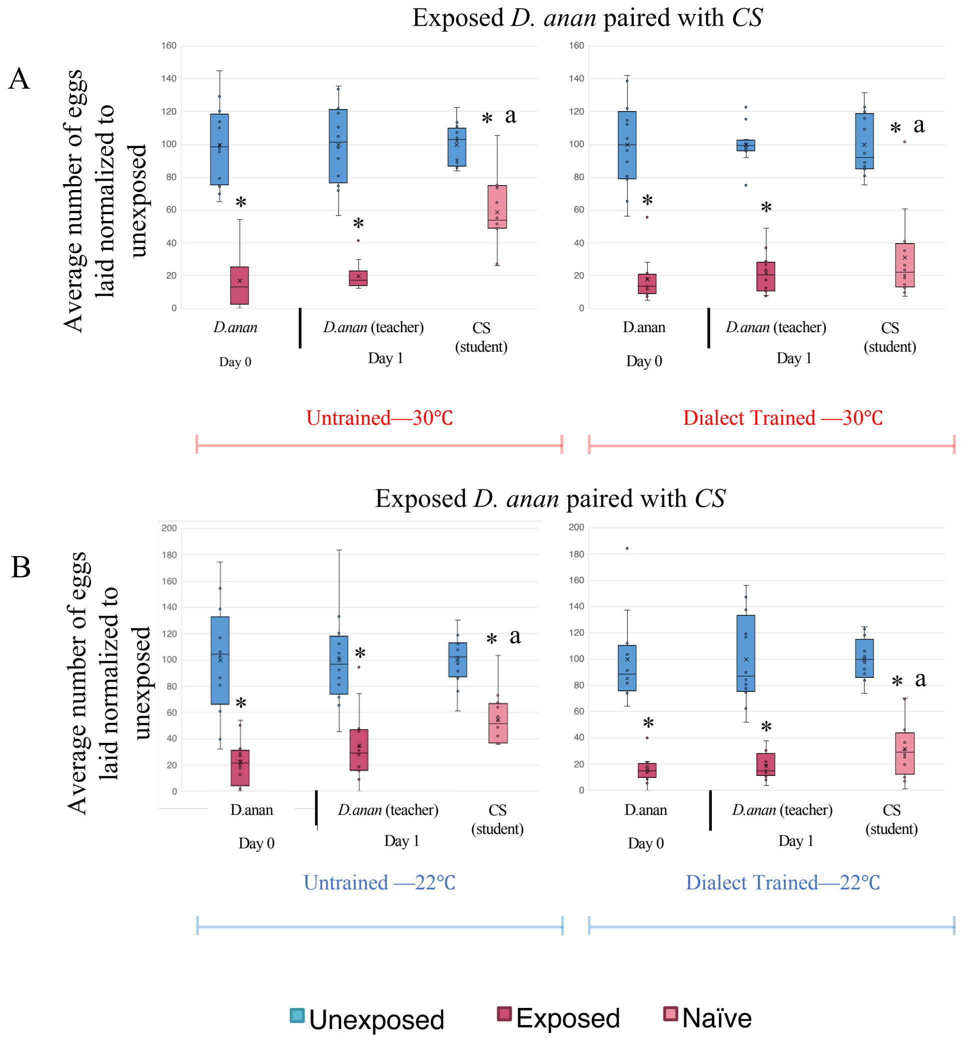

Exposed D. anan paired with CS
A
Average number of eggs laid normalized to unexposed
D.anan
Day 0
D.anan (teacher)
Day 1
CS (student)
Untrained—30°C
Dialect Trained —30°C
Exposed D. anan paired with CS
B
Untrained —22°C
Dialect Trained—22°C
Unexposed
Exposed
Naïve

Supplementary Figure 2. The optic lobe is required for dialect training shown with R10B01 driver.

(A) Cartoon schematic of the region of interest, the optic lobe. (B) Confocal image of adult brain where R10B01^{GAL4} is driving UAS-CD8-GFP, stained with nc82 (magenta) and DAPI (teal). Fly light intensity/distribution score is 5/2 and was the only region of the brain identified. Percentage of eggs laid by exposed flies normalized to eggs laid by unexposed flies is shown. UAS-shi^{ts} crossed to R10B01^{GAL4} trained by *D. ananassae* at the permissive temperature shows wild-type trained state (C), but at the restrictive temperature shows defective acquisition in the trained state (D). Blue lines indicate permissive temperature, while red lines indicate restrictive temperature. Trained and untrained states are labeled. Plots are standard Tukey Box Plots, where the bounds of the shaded box represent the first and third quartile; whiskers indicate the minimum and maximum data point within 1.5 times the interquartile range. Mean is denoted with an 'X.' Data used to generate the plots have been superimposed on the graph as open circles (n = 12 biologically independent experiments) (*$p < 0.05$, a→ $p < 0.05$ comparing trained and untrained students within a given temperature when both restrictive and permissive temperatures are tested, b→ p = ns comparing trained and untrained students within a given temperature when both restrictive and permissive temperatures are tested).

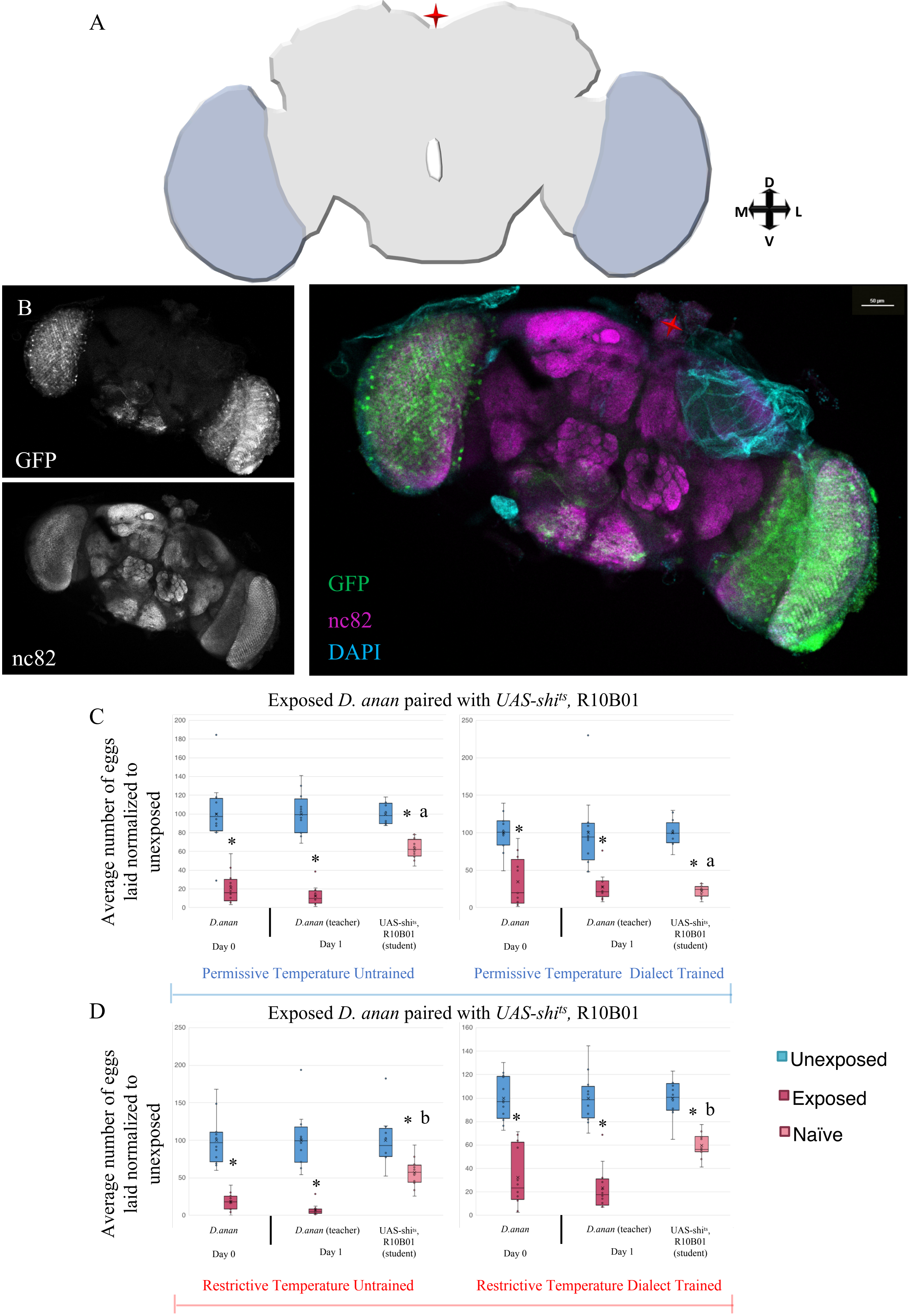

A
D
M
L
V
B
GFP
nc82
GFP
nc82
DAPI
50 µm
C
Exposed D. anan paired with UAS-shits, R10B01
Average number of eggs laid normalized to unexposed
D.anan
Day 0
D.anan (teacher)
Day 1
UAS-shits, R10B01 (student)
Permissive Temperature Untrained
Permissive Temperature Dialect Trained
D
Exposed D. anan paired with UAS-shits, R10B01
Restrictive Temperature Untrained
Restrictive Temperature Dialect Trained
Unexposed
Exposed
Naïve

Supplementary Figure 3. The optic lobe is required for dialect training shown with R17F12 driver.

(A) Cartoon schematic of the region of interest, the optic lobe. (B) Confocal image of adult brain where $R17F12^{GAL4}$ is driving UAS-CD8-GFP, stained with nc82 (magenta) and DAPI (teal). Fly light intensity/distribution score is 5/2 and was the only region of the brain identified. Percentage of eggs laid by exposed flies normalized to eggs laid by unexposed flies is shown. $UAS\text{-}shi^{ts}$ crossed to $R17F12^{GAL4}$ trained by *D. ananassae* at the permissive temperature shows wild-type trained state (C), but at the restrictive temperature shows defective acquisition in the trained state (D). Blue lines indicate permissive temperature, while red lines indicate restrictive temperature. Trained and untrained states are labeled. Plots are standard Tukey Box Plots, where the bounds of the shaded box represent the first and third quartile; whiskers indicate the minimum and maximum data point within 1.5 times the interquartile range. Mean is denoted with an 'X.' Data used to generate the plots have been superimposed on the graph as open circles (n = 12 biologically independent experiments) (*$p < 0.05$, a→ $p < 0.05$ comparing trained and untrained students within a given temperature when both restrictive and permissive temperatures are tested, b→ p = ns comparing trained and untrained students within a given temperature when both restrictive and permissive temperatures are tested).

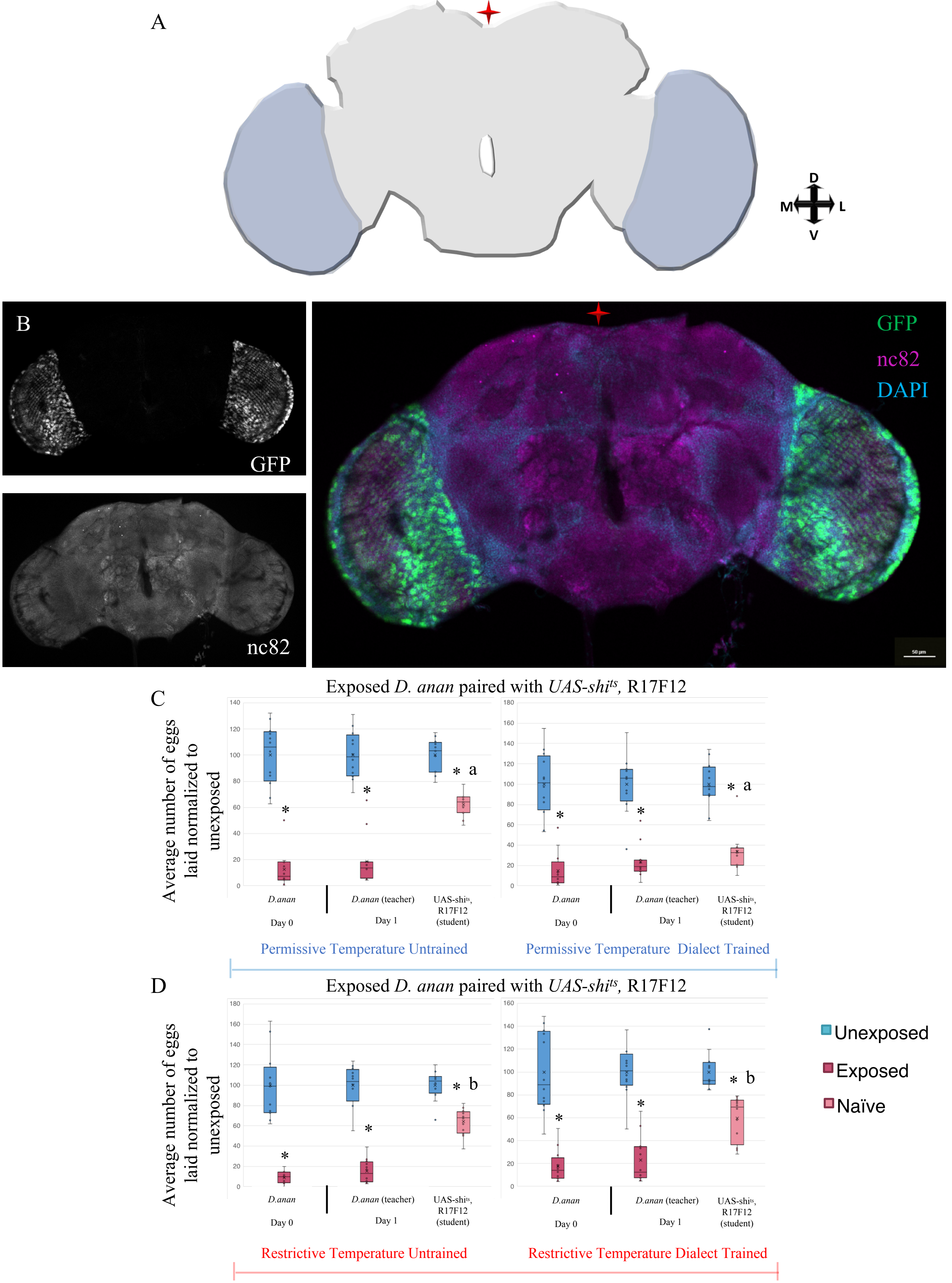

A
D
M
L
V
B
GFP
nc82
GFP
nc82
DAPI
C
Exposed D. anan paired with UAS-shits, R17F12
Average number of eggs laid normalized to unexposed
D.anan
Day 0
D.anan (teacher)
Day 1
UAS-shits, R17F12 (student)
Permissive Temperature Untrained
Permissive Temperature Dialect Trained
D
Exposed D. anan paired with UAS-shits, R17F12
Average number of eggs laid normalized to unexposed
D.anan
Day 0
D.anan (teacher)
Day 1
UAS-shits, R17F12 (student)
Restrictive Temperature Untrained
Restrictive Temperature Dialect Trained
Unexposed
Exposed
Naïve

Supplementary Figure 4. The mushroom body is required for dialect training shown with R13F02 driver.

(A) Cartoon schematic of the region of interest, the mushroom body. (B) Confocal image of adult brain where R13F02^{GAL4} is driving UAS-CD8-GFP, stained with nc82 (magenta) and DAPI (teal). Fly light intensity/distribution score is 5/4 and was the only region of the brain identified. Percentage of eggs laid by exposed flies normalized to eggs laid by unexposed flies is shown. UAS-shi^{ts} crossed to R13F02^{GAL4} trained by *D. ananassae* at the permissive temperature shows wild-type trained state (C), but at the restrictive temperature shows defective acquisition in the trained state (D). Blue lines indicate permissive temperature, while red lines indicate restrictive temperature. Trained and untrained states are labeled. Plots are standard Tukey Box Plots, where the bounds of the shaded box represent the first and third quartile; whiskers indicate the minimum and maximum data point within 1.5 times the interquartile range. Mean is denoted with an 'X.' Data used to generate the plots have been superimposed on the graph as open circles (n = 12 biologically independent experiments) (*p < 0.05, a→ p < 0.05 comparing trained and untrained students within a given temperature when both restrictive and permissive temperatures are tested, b→ p = ns comparing trained and untrained students within a given temperature when both restrictive and permissive temperatures are tested).

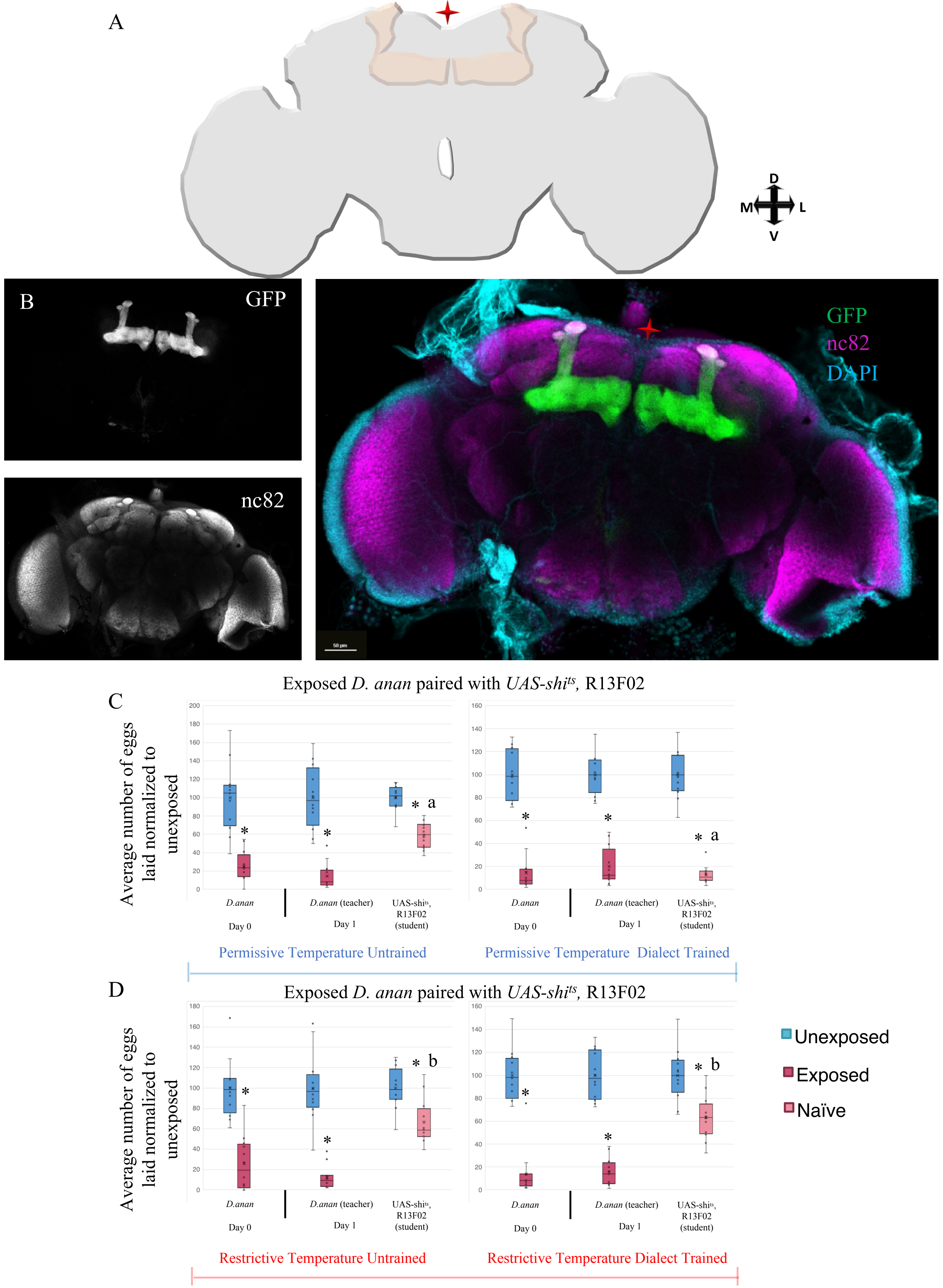
A
D
M
L
V
B
GFP
nc82
GFP
nc82
DAPI
C
Exposed D. anan paired with UAS-shi^ts, R13F02
Average number of eggs laid normalized to unexposed
* a
* a
D.anan
Day 0
D.anan (teacher)
Day 1
UAS-shi^ts, R13F02 (student)
Permissive Temperature Untrained
Permissive Temperature Dialect Trained
D
Exposed D. anan paired with UAS-shi^ts, R13F02
Average number of eggs laid normalized to unexposed
* b
* b
Restrictive Temperature Untrained
Restrictive Temperature Dialect Trained
Unexposed
Exposed
Naïve

Supplementary Figure 5. The mushroom body is required for dialect training shown with R16A06 driver.

(A) Cartoon schematic of the region of interest, the mushroom body. (B) Confocal image of adult brain where $R16A06^{GAL4}$ is driving UAS-CD8-GFP, stained with nc82 (magenta) and DAPI (teal). Percentage of eggs laid by exposed flies normalized to eggs laid by unexposed flies is shown. UAS-shi^{ts} crossed to $R16A06^{GAL4}$ trained by *D. ananassae* at the permissive temperature shows wild-type trained state (C), but at the restrictive temperature shows defective acquisition in the trained state (D). Blue lines indicate permissive temperature, while red lines indicate restrictive temperature. Trained and untrained states are labeled. Plots are standard Tukey Box Plots, where the bounds of the shaded box represent the first and third quartile; whiskers indicate the minimum and maximum data point within 1.5 times the interquartile range. Mean is denoted with an 'X.' Data used to generate the plots have been superimposed on the graph as open circles ($n = 12$ biologically independent experiments) (*$p < 0.05$, a→ $p < 0.05$ comparing trained and untrained students within a given temperature when both restrictive and permissive temperatures are tested, b→ p = ns comparing trained and untrained students within a given temperature when both restrictive and permissive temperatures are tested).

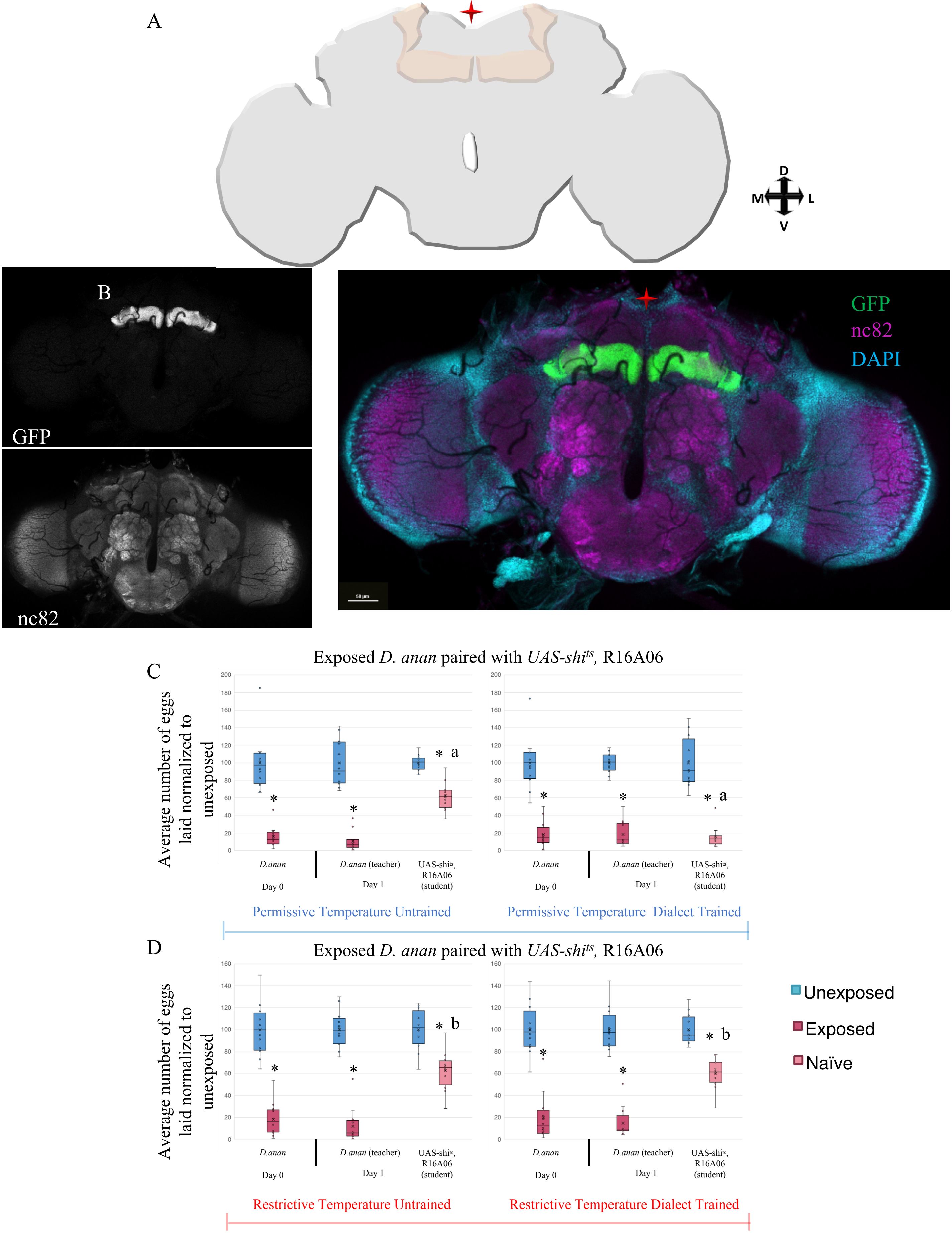
A
D
M
L
V
B
GFP
nc82
GFP
nc82
DAPI
50 µm
C
Exposed D. anan paired with UAS-shi^ts, R16A06
Average number of eggs laid normalized to unexposed
D.anan
Day 0
D.anan (teacher)
Day 1
UAS-shits, R16A06 (student)
* a
Permissive Temperature Untrained
Permissive Temperature Dialect Trained
D
Exposed D. anan paired with UAS-shi^ts, R16A06
* b
Restrictive Temperature Untrained
Restrictive Temperature Dialect Trained
Unexposed
Exposed
Naïve

Supplementary Figure 6. The antennal lobe is required for dialect training shown with R21C11 driver.

(A) Cartoon schematic of the region of interest, the antennal lobe. (B) Confocal image of adult brain where R21C11^{GAL4} is driving UAS-CD8-GFP, stained with nc82 (magenta) and DAPI (teal). Fly light intensity/distribution score is 4/4 and was the only region of the brain identified. Percentage of eggs laid by exposed flies normalized to eggs laid by unexposed flies is shown. UAS-shi^{ts} crossed to R21C11^{GAL4} trained by *D. ananassae* at the permissive temperature shows wild-type trained state (C), but at the restrictive temperature shows defective acquisition in the trained state (D). Blue lines indicate permissive temperature, while red lines indicate restrictive temperature. Trained and untrained states are labeled under each graph. Blue lines indicate permissive temperature, while red lines indicate restrictive temperature. Trained and untrained states are labeled. Plots are standard Tukey Box Plots, where the bounds of the shaded box represent the first and third quartile; whiskers indicate the minimum and maximum data point within 1.5 times the interquartile range. Mean is denoted with an 'X.' Data used to generate the plots have been superimposed on the graph as open circles (n = 12 biologically independent experiments) (*$p < 0.05$, a→ $p < 0.05$ comparing trained and untrained students within a given temperature when both restrictive and permissive temperatures are tested, b→ p = ns comparing trained and untrained students within a given temperature when both restrictive and permissive temperatures are tested).

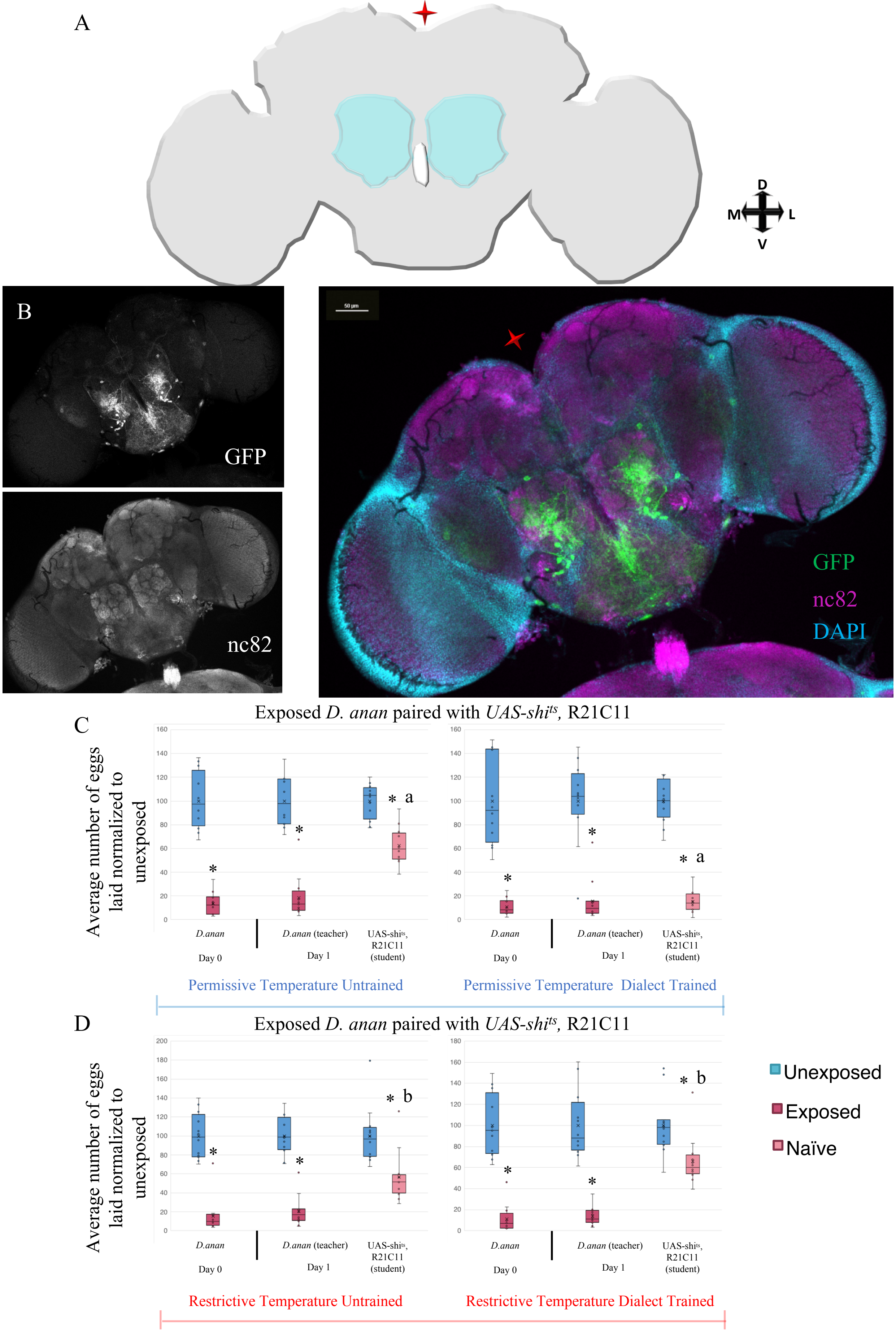
A
D
M
L
V
B
GFP
nc82
50 μm
GFP
nc82
DAPI
C
Exposed D. anan paired with UAS-shi^ts, R21C11
Average number of eggs laid normalized to unexposed
* a
D.anan
Day 0
D.anan (teacher)
Day 1
UAS-shi^ts, R21C11 (student)
Permissive Temperature Untrained
Permissive Temperature Dialect Trained
D
Exposed D. anan paired with UAS-shi^ts, R21C11
Average number of eggs laid normalized to unexposed
* b
Restrictive Temperature Untrained
Restrictive Temperature Dialect Trained
Unexposed
Exposed
Naïve

Supplementary Figure 7. The antennal lobe is required for dialect training shown with R14H04 driver.

(A) Cartoon schematic of the region of interest, the antennal lobe. (B) Confocal image of adult brain where R14H04^{GAL4} is driving UAS-CD8-GFP, stained with nc82 (magenta) and DAPI (teal). Fly light intensity/distribution score is 5/5 and was the only region of the brain identified. Percentage of eggs laid by exposed flies normalized to eggs laid by unexposed flies is shown. UAS-shi^{ts} crossed to R14H04^{GAL4} trained by *D. ananassae* at the permissive temperature shows wild-type trained state (C), but at the restrictive temperature shows defective acquisition in the trained state (D). Blue lines indicate permissive temperature, while red lines indicate restrictive temperature. Trained and untrained states are labeled under each graph. Blue lines indicate permissive temperature, while red lines indicate restrictive temperature. Trained and untrained states are labeled. Plots are standard Tukey Box Plots, where the bounds of the shaded box represent the first and third quartile; whiskers indicate the minimum and maximum data point within 1.5 times the interquartile range. Mean is denoted with an 'X.' Data used to generate the plots have been superimposed on the graph as open circles (n = 12 biologically independent experiments) (*$p < 0.05$, a→ $p < 0.05$ comparing trained and untrained students within a given temperature when both restrictive and permissive temperatures are tested, b→ p = ns comparing trained and untrained students within a given temperature when both restrictive and permissive temperatures are tested).

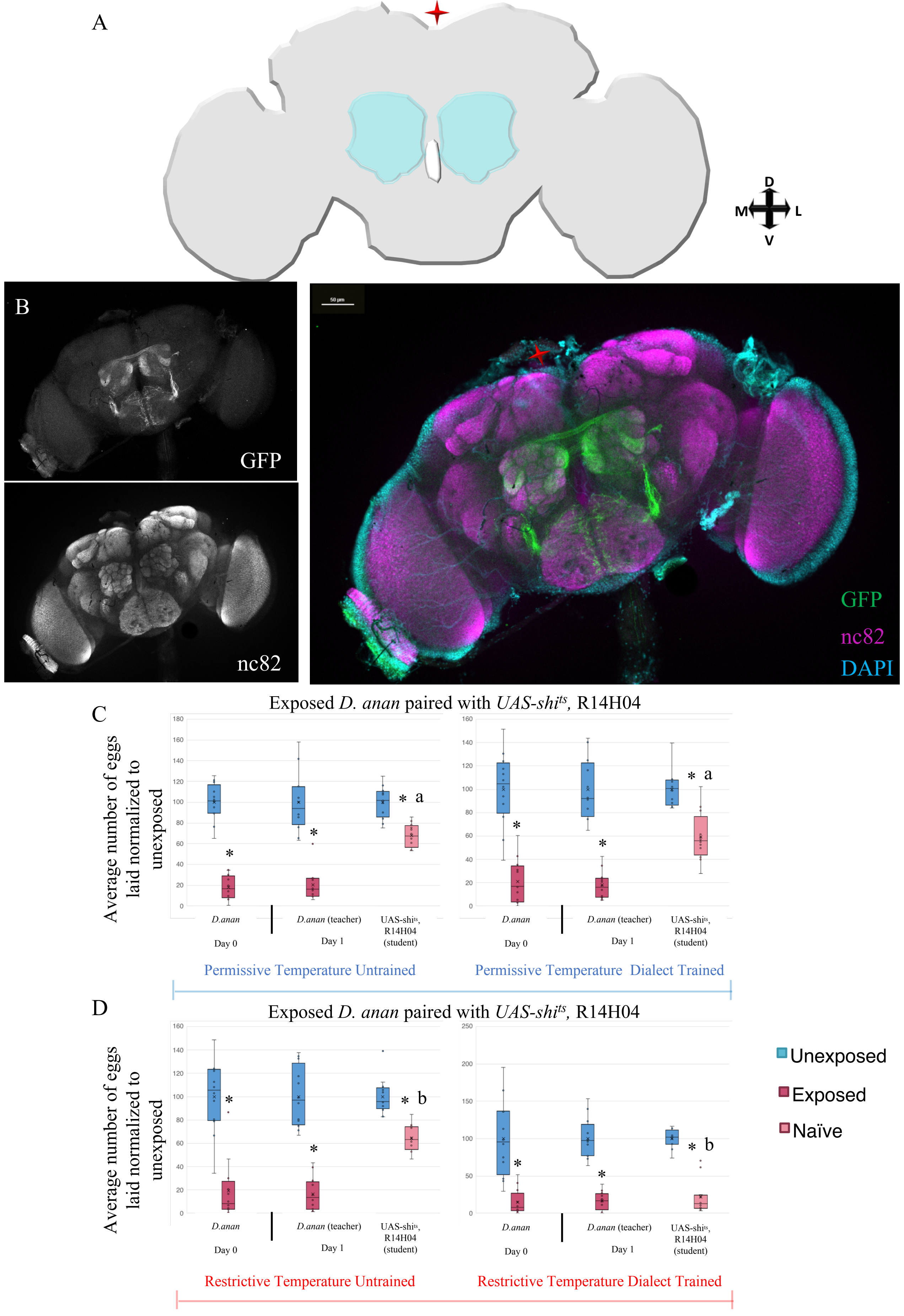
A
D
M
L
V
B
GFP
nc82
50 µm
GFP
nc82
DAPI
C
Exposed D. anan paired with UAS-shi^ts, R14H04
Average number of eggs laid normalized to unexposed
D.anan
Day 0
D.anan (teacher)
Day 1
UAS-shits, R14H04 (student)
Permissive Temperature Untrained
Permissive Temperature Dialect Trained
D
Exposed D. anan paired with UAS-shi^ts, R14H04
Average number of eggs laid normalized to unexposed
Restrictive Temperature Untrained
Restrictive Temperature Dialect Trained
Unexposed
Exposed
Naïve

Supplementary Figure 8. The lateral horn is required for dialect training shown with R10G01 driver.

(A) Cartoon schematic of the region of interest, the lateral horn. (B) Confocal image of adult brain where R10G01^{GAL4} is driving UAS-CD8-GFP, stained with nc82 (magenta) and DAPI (teal). Fly light intensity/distribution score is 2/2 and was the only region of the brain identified. Percentage of eggs laid by exposed flies normalized to eggs laid by unexposed flies is shown. UAS-shi^{ts} crossed to R10G01^{GAL4} trained by *D. ananassae* at the permissive temperature shows wild-type trained state (C), but at the restrictive temperature shows defective acquisition in the trained state (D). Blue lines indicate permissive temperature, while red lines indicate restrictive temperature. Trained and untrained states are labeled. Plots are standard Tukey Box Plots, where the bounds of the shaded box represent the first and third quartile; whiskers indicate the minimum and maximum data point within 1.5 times the interquartile range. Mean is denoted with an 'X.' Data used to generate the plots have been superimposed on the graph as open circles (n = 12 biologically independent experiments) (*$p < 0.05$, a→ $p < 0.05$ comparing trained and untrained students within a given temperature when both restrictive and permissive temperatures are tested, b→ p = ns comparing trained and untrained students within a given temperature when both restrictive and permissive temperatures are tested).

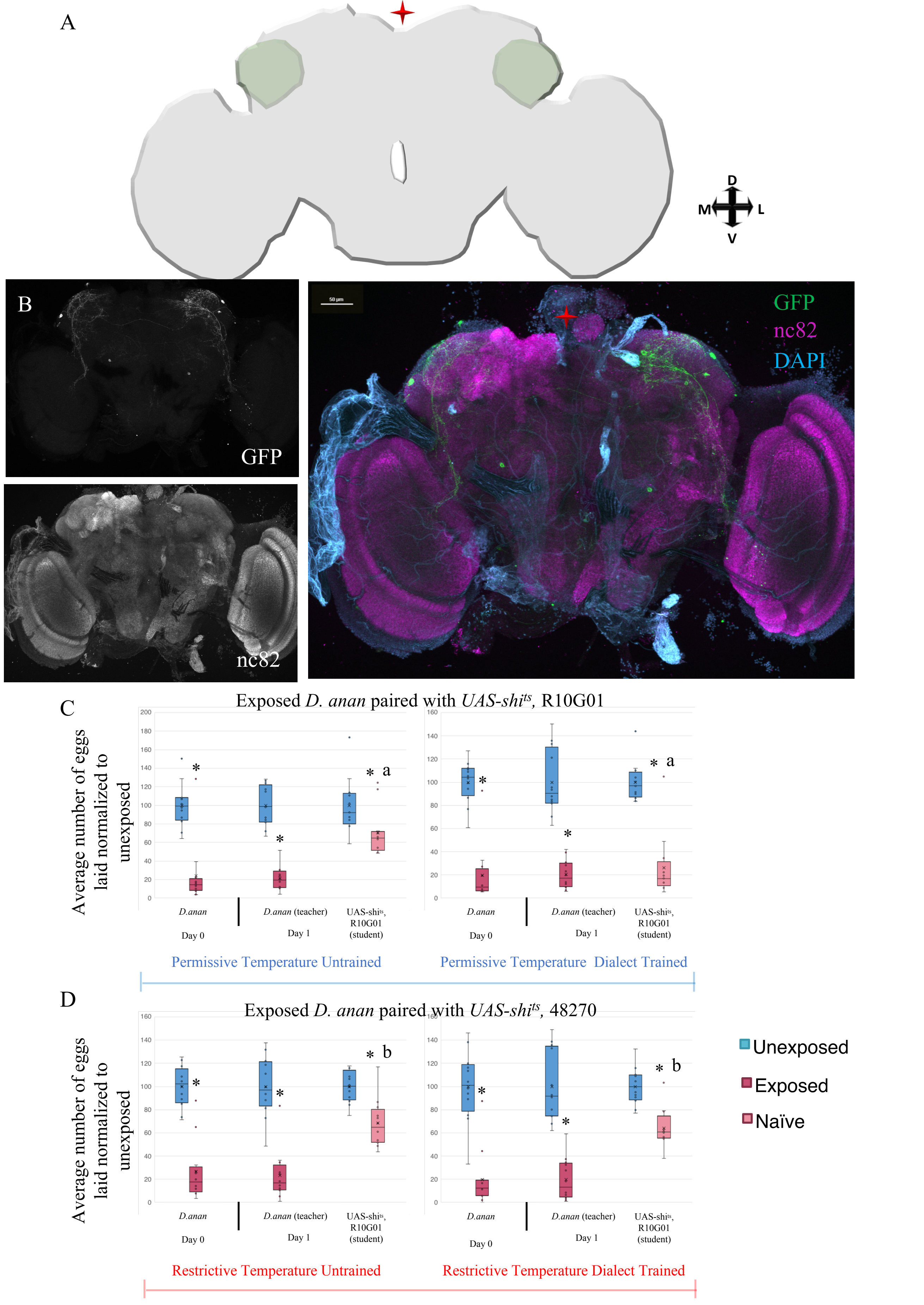
A
D
M
L
V
B
GFP
nc82
50 µm
GFP
nc82
DAPI
C
Exposed D. anan paired with UAS-shi^ts, R10G01
Average number of eggs laid normalized to unexposed
D.anan
Day 0
D.anan (teacher)
Day 1
UAS-shi^ts, R10G01 (student)
Permissive Temperature Untrained
Permissive Temperature Dialect Trained
D
Exposed D. anan paired with UAS-shi^ts, 48270
Restrictive Temperature Untrained
Restrictive Temperature Dialect Trained
Unexposed
Exposed
Naïve

Supplementary Figure 9. The fan-shaped body is required for dialect training shown with R75G12 driver.

(A) Cartoon schematic of the region of interest, the fan-shaped body. (B) Confocal image of adult brain where $R75G12^{GAL4}$ is driving UAS-CD8-GFP, stained with nc82 (magenta) and DAPI (teal). Fly light intensity/distribution score is 5/3 and was the only region of the brain identified. Percentage of eggs laid by exposed flies normalized to eggs laid by unexposed flies is shown. UAS-shi^{ts} crossed to $R75G12^{GAL4}$ trained by *D. ananassae* at the permissive temperature shows wild-type trained state (C), but at the restrictive temperature shows defective acquisition in the trained state (D). Blue lines indicate permissive temperature, while red lines indicate restrictive temperature. Trained and untrained states are labeled. Plots are standard Tukey Box Plots, where the bounds of the shaded box represent the first and third quartile; whiskers indicate the minimum and maximum data point within 1.5 times the interquartile range. Mean is denoted with an 'X.' Data used to generate the plots have been superimposed on the graph as open circles ($n = 12$ biologically independent experiments) (*$p < 0.05$, a→ $p < 0.05$ comparing trained and untrained students within a given temperature when both restrictive and permissive temperatures are tested, b→ p = ns comparing trained and untrained students within a given temperature when both restrictive and permissive temperatures are tested).

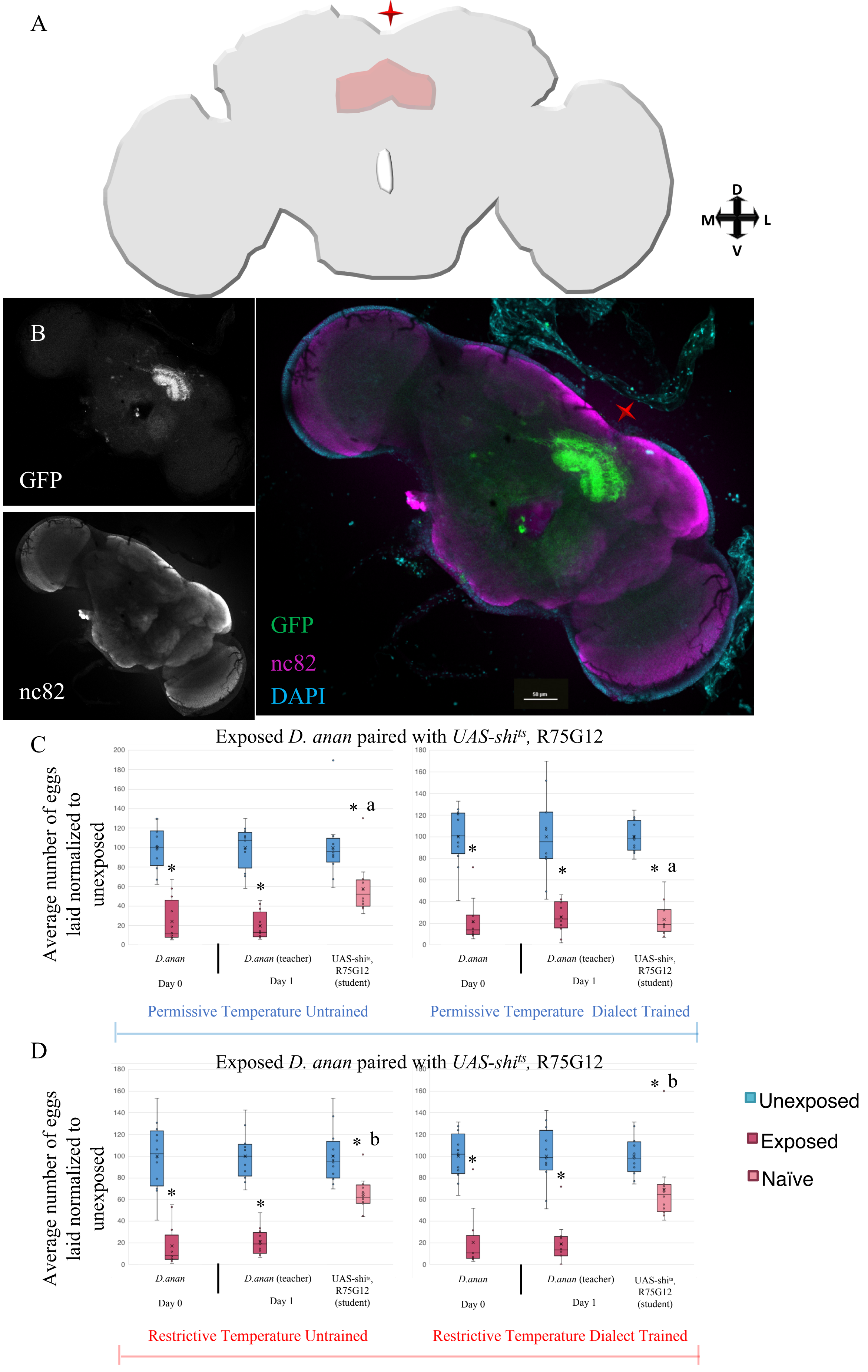
A
D
M
L
V
B
GFP
nc82
GFP
nc82
DAPI
50 µm
C
Exposed D. anan paired with UAS-shi^ts, R75G12
Average number of eggs laid normalized to unexposed
D.anan
Day 0
D.anan (teacher)
Day 1
UAS-shi^ts, R75G12 (student)
* a
Permissive Temperature Untrained
Permissive Temperature Dialect Trained
D
Exposed D. anan paired with UAS-shi^ts, R75G12
Average number of eggs laid normalized to unexposed
* b
Restrictive Temperature Untrained
Restrictive Temperature Dialect Trained
Unexposed
Exposed
Naïve

Supplementary Figure 10. The ellipsoid body is required for dialect training shown with R11F03 driver.

(A) Cartoon schematic of the region of interest, the ellipsoid body. (B) Confocal image of adult brain where $R11F03^{GAL4}$ is driving UAS-CD8-GFP, stained with nc82 (magenta) and DAPI (teal). Fly light intensity/distribution score is 3/5 and was the only region of the brain identified. Percentage of eggs laid by exposed flies normalized to eggs laid by unexposed flies is shown. UAS-shi^{ts} crossed to $R11F03^{GAL4}$ trained by *D. ananassae* at the permissive temperature shows wild-type trained state (C), but at the restrictive temperature shows defective acquisition in the trained state (D). Blue lines indicate permissive temperature, while red lines indicate restrictive temperature. Trained and untrained states are labeled. Plots are standard Tukey Box Plots, where the bounds of the shaded box represent the first and third quartile; whiskers indicate the minimum and maximum data point within 1.5 times the interquartile range. Mean is denoted with an 'X.' Data used to generate the plots have been superimposed on the graph as open circles (n = 12 biologically independent experiments) (*$p < 0.05$, a→ $p < 0.05$ comparing trained and untrained students within a given temperature when both restrictive and permissive temperatures are tested, b→ p = ns comparing trained and untrained students within a given temperature when both restrictive and permissive temperatures are tested).

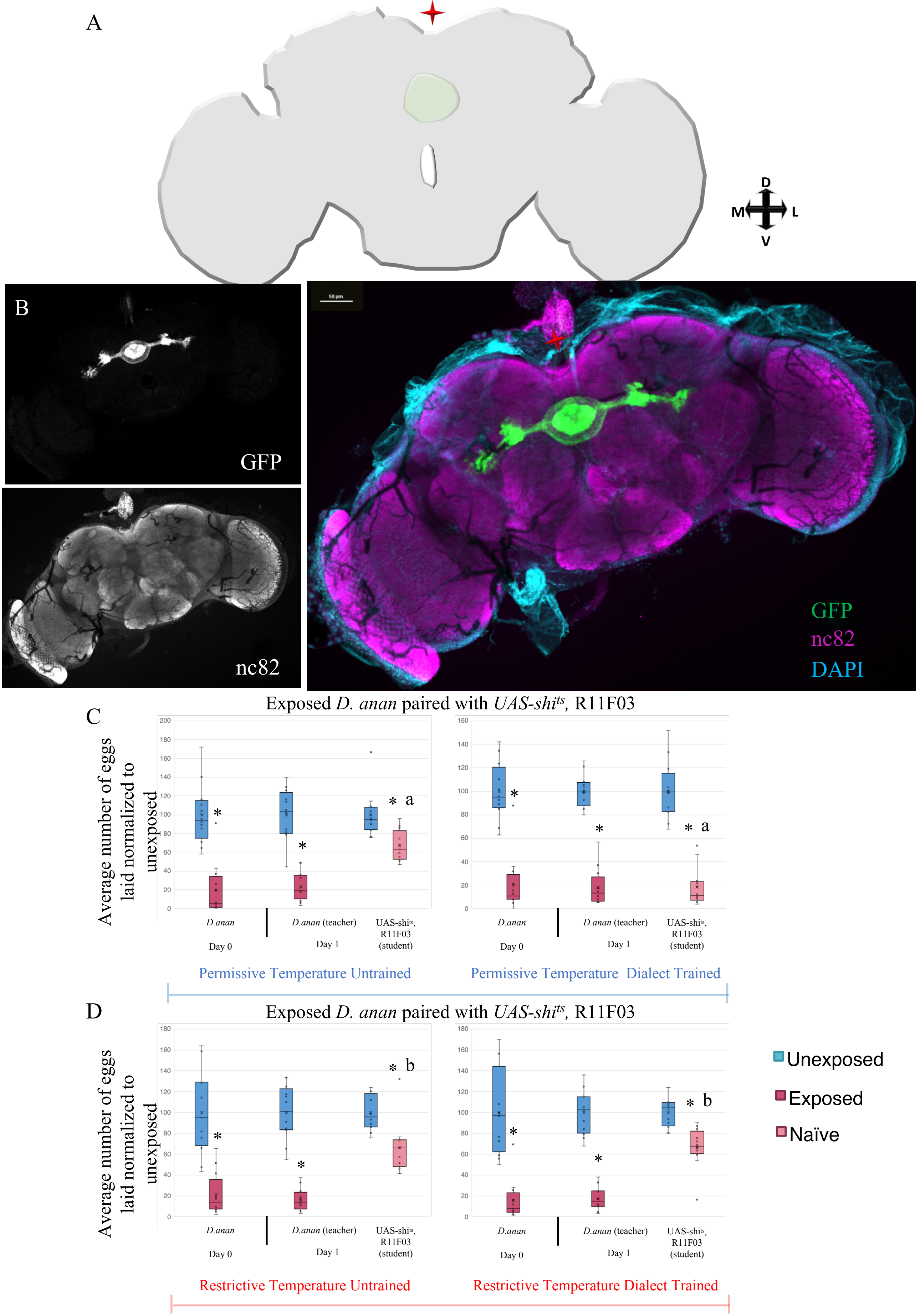

A
D
M
L
V
B
GFP
nc82
50 µm
GFP
nc82
DAPI
C
Exposed D. anan paired with UAS-shits, R11F03
Average number of eggs laid normalized to unexposed
D.anan
Day 0
D.anan (teacher)
UAS-shits, R11F03 (student)
Day 1
Permissive Temperature Untrained
Permissive Temperature Dialect Trained
D
Exposed D. anan paired with UAS-shits, R11F03
Average number of eggs laid normalized to unexposed
Restrictive Temperature Untrained
Restrictive Temperature Dialect Trained
Unexposed
Exposed
Naïve

Supplementary Figure 11. The ellipsoid body is required for dialect training shown with R15F02 driver.

(A) Cartoon schematic of the region of interest, the ellipsoid body. (B) Confocal image of adult brain where $R15F02^{GAL4}$ is driving UAS-CD8-GFP, stained with nc82 (magenta) and DAPI (teal). Fly light intensity/distribution score is 5/5 and was the only region of the brain identified. Percentage of eggs laid by exposed flies normalized to eggs laid by unexposed flies is shown. UAS-shi^{ts} crossed to $R15F02^{GAL4}$ trained by *D. ananassae* at the permissive temperature shows wild-type trained state (C), but at the restrictive temperature shows defective acquisition in the trained state (D). Blue lines indicate permissive temperature, while red lines indicate restrictive temperature. Trained and untrained states are labeled. Plots are standard Tukey Box Plots, where the bounds of the shaded box represent the first and third quartile; whiskers indicate the minimum and maximum data point within 1.5 times the interquartile range. Mean is denoted with an 'X.' Data used to generate the plots have been superimposed on the graph as open circles (n = 12 biologically independent experiments) (*$p < 0.05$, a→ $p < 0.05$ comparing trained and untrained students within a given temperature when both restrictive and permissive temperatures are tested, b→ p = ns comparing trained and untrained students within a given temperature when both restrictive and permissive temperatures are tested).

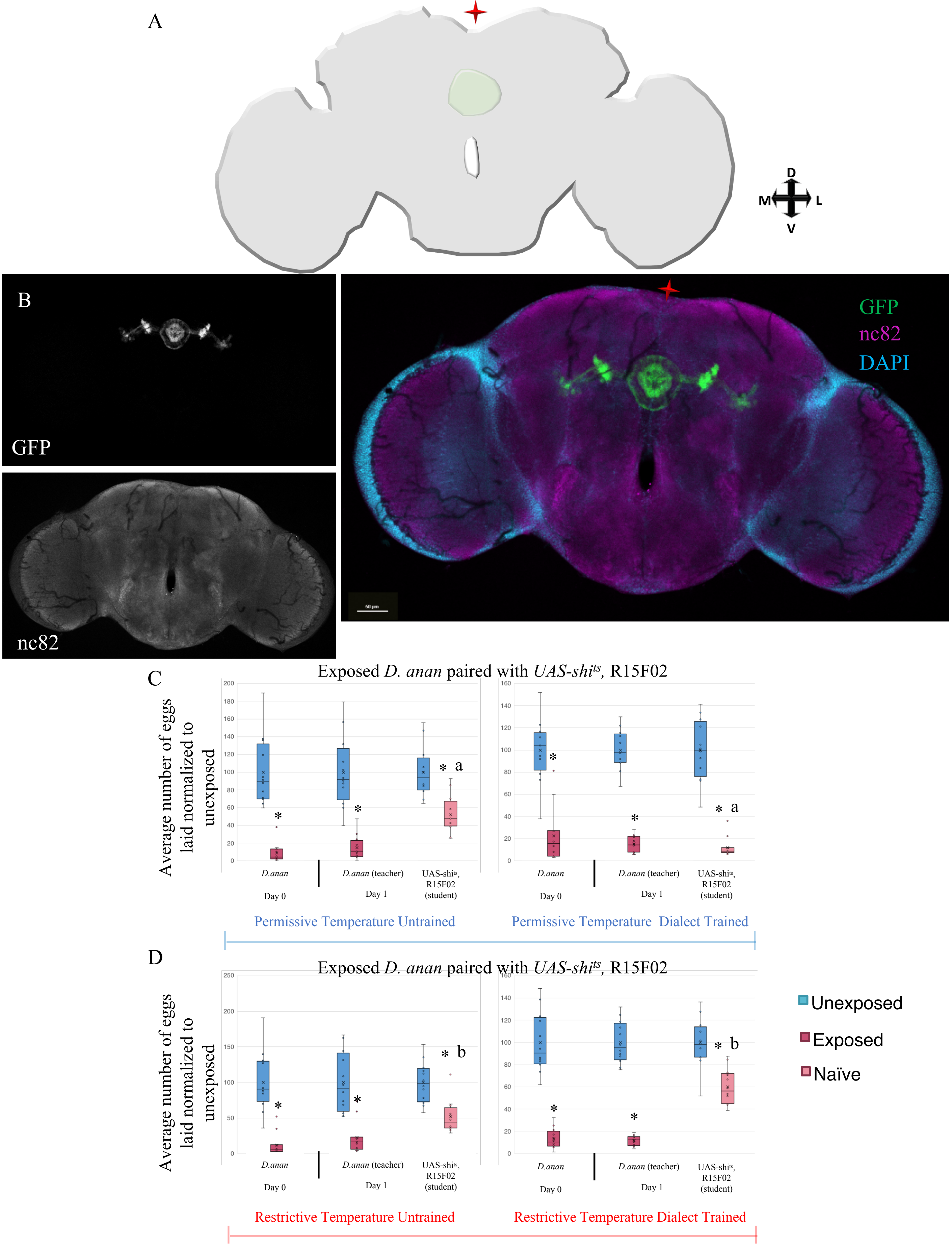
A
D
M
L
V
B
GFP
nc82
GFP
nc82
DAPI
50 µm
C
Exposed D. anan paired with UAS-shits, R15F02
Average number of eggs laid normalized to unexposed
D.anan
Day 0
D.anan (teacher)
Day 1
UAS-shits, R15F02 (student)
Permissive Temperature Untrained
Permissive Temperature Dialect Trained
D
Exposed D. anan paired with UAS-shits, R15F02
Average number of eggs laid normalized to unexposed
Restrictive Temperature Untrained
Restrictive Temperature Dialect Trained
Unexposed
Exposed
Naïve

Supplementary Figure 12. The bulb is dispensable for dialect training shown with R32B03 driver.

(A) Cartoon schematic of the region of interest, the bulb. (B) Confocal image of adult brain where $R32B03^{GAL4}$ is driving UAS-CD8-GFP, stained with nc82 (magenta) and DAPI (teal). Fly light intensity/distribution score is 5/4 and was the only region of the brain identified. Percentage of eggs laid by exposed flies normalized to eggs laid by unexposed flies is shown. Percentage of eggs laid by exposed flies normalized to eggs laid by unexposed flies is shown. UAS-shi^{ts} crossed to $R32B03^{GAL4}$ trained by *D. ananassae* at both the permissive temperature (C) and restrictive temperature (D) shows wild-type trained state. Blue lines indicate permissive temperature, while red lines indicate restrictive temperature. Trained and untrained states are labeled. Plots are standard Tukey Box Plots, where the bounds of the shaded box represent the first and third quartile; whiskers indicate the minimum and maximum data point within 1.5 times the interquartile range. Mean is denoted with an 'X.' Data used to generate the plots have been superimposed on the graph as open circles (n = 12 biologically independent experiments) (*$p < 0.05$, a→ $p < 0.05$ comparing trained and untrained students within a given temperature when both restrictive and permissive temperatures are tested, b→ p = ns comparing trained and untrained students within a given temperature when both restrictive and permissive temperatures are tested).

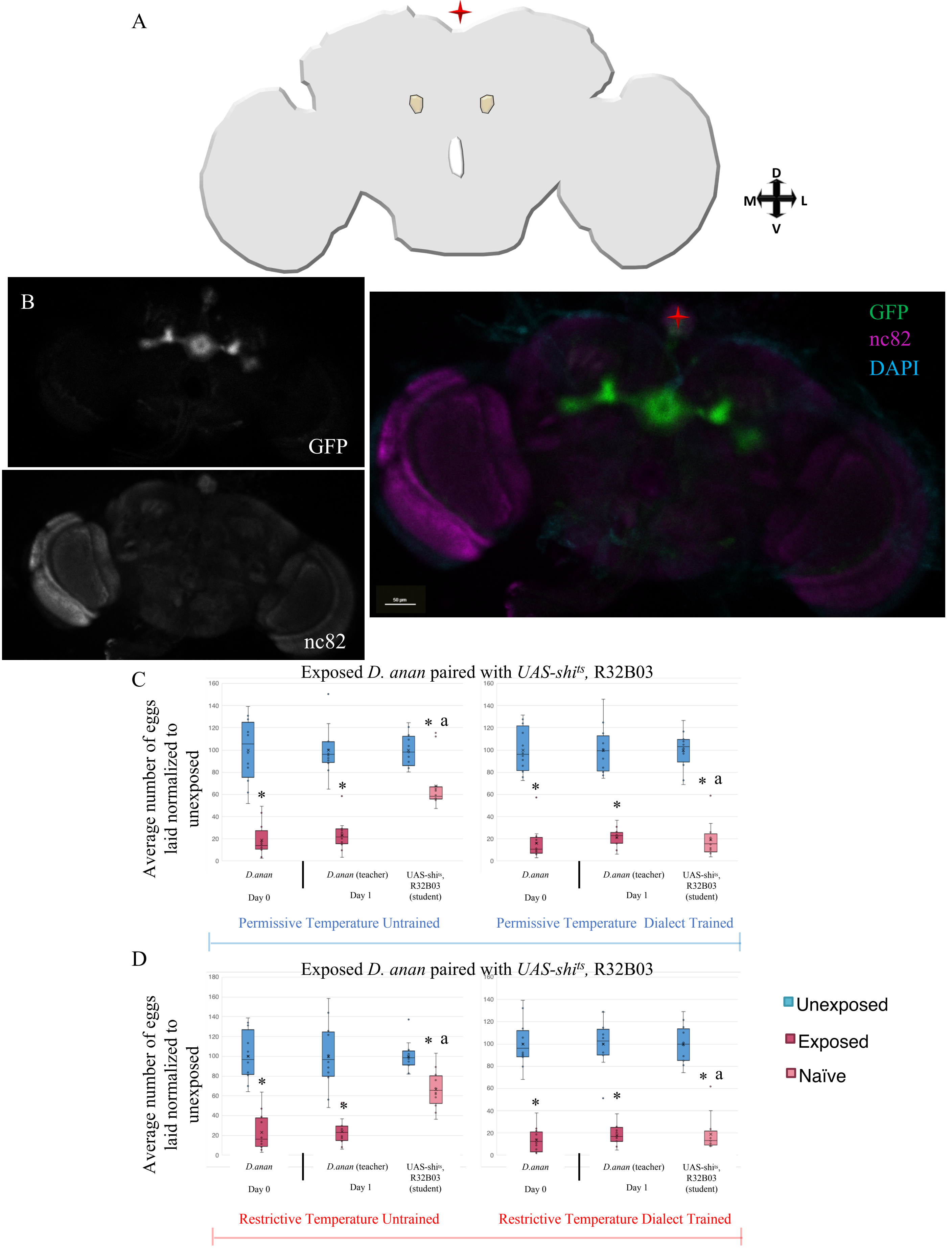
A
D
M
L
V
B
GFP
nc82
GFP
nc82
DAPI
C
Exposed D. anan paired with UAS-shits, R32B03
Average number of eggs laid normalized to unexposed
D.anan
Day 0
D.anan (teacher)
Day 1
UAS-shits, R32B03 (student)
* a
Permissive Temperature Untrained
Permissive Temperature Dialect Trained
D
Exposed D. anan paired with UAS-shits, R32B03
Average number of eggs laid normalized to unexposed
Restrictive Temperature Untrained
Restrictive Temperature Dialect Trained
Unexposed
Exposed
Naïve

Supplementary Figure 13. The prow is dispensable for dialect training shown with R25G04 driver.

(A) Cartoon schematic of the region of interest, the prow. (B) Confocal image of adult brain where $R25G04^{GAL4}$ is driving UAS-CD8-GFP, stained with nc82 (magenta) and DAPI (teal). Fly light intensity/distribution score is 4/4 and was the only region of the brain identified. Percentage of eggs laid by exposed flies normalized to eggs laid by unexposed flies is shown. UAS-shi^{ts} crossed to $R25G04^{GAL4}$ trained by *D. ananassae* at both the permissive temperature (C) and restrictive temperature (D) shows wild-type trained state. Blue lines indicate permissive temperature, while red lines indicate restrictive temperature. Trained and untrained states are labeled. Plots are standard Tukey Box Plots, where the bounds of the shaded box represent the first and third quartile; whiskers indicate the minimum and maximum data point within 1.5 times the interquartile range. Mean is denoted with an 'X.' Data used to generate the plots have been superimposed on the graph as open circles (n = 12 biologically independent experiments) (*$p < 0.05$, a→ $p < 0.05$ comparing trained and untrained students within a given temperature when both restrictive and permissive temperatures are tested, b→ p = ns comparing trained and untrained students within a given temperature when both restrictive and permissive temperatures are tested).

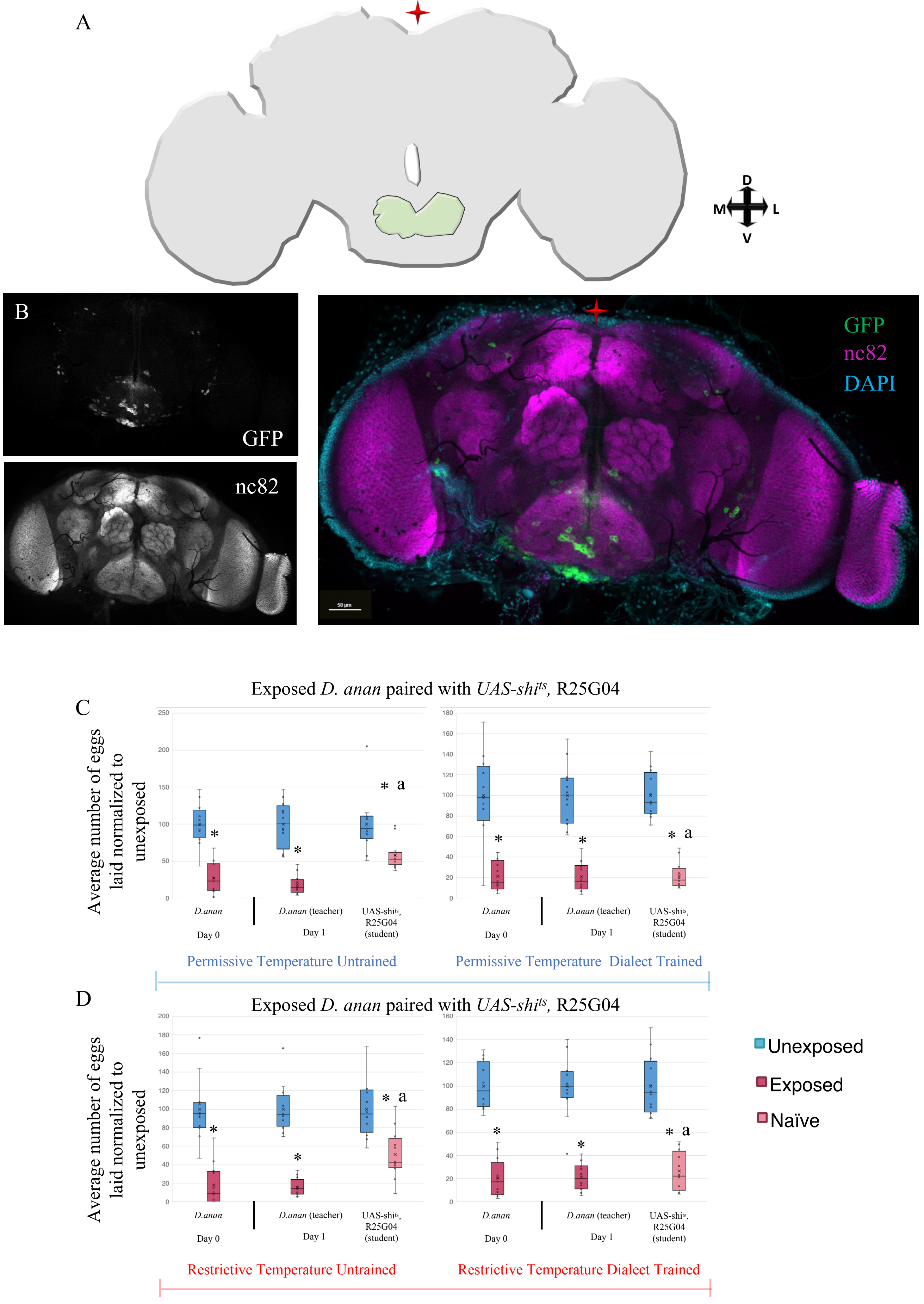

A
D
M
L
V
B
GFP
nc82
GFP
nc82
DAPI
C
Exposed D. anan paired with UAS-shi^ts, R25G04
Average number of eggs laid normalized to unexposed
D.anan
Day 0
D.anan (teacher)
Day 1
UAS-shits, R25G04 (student)
Permissive Temperature Untrained
Permissive Temperature Dialect Trained
D
Exposed D. anan paired with UAS-shi^ts, R25G04
Restrictive Temperature Untrained
Restrictive Temperature Dialect Trained
Unexposed
Exposed
Naïve

Supplementary Figure 14. The superior clamp is dispensable for dialect training shown with R30F03 driver.

(A) Cartoon schematic of the region of interest, the superior clamp. (B) Confocal image of adult brain where R30F03^{GAL4} is driving UAS-CD8-GFP, stained with nc82 (magenta) and DAPI (teal). Fly light intensity/distribution score is 3/3 and was the only region of the brain identified. Percentage of eggs laid by exposed flies normalized to eggs laid by unexposed flies is shown. UAS-shi^{ts} crossed to R30F03^{GAL4} trained by *D. ananassae* at both the permissive temperature (C) and restrictive temperature (D) shows wild-type trained state. Blue lines indicate permissive temperature, while red lines indicate restrictive temperature. Trained and untrained states are labeled. Plots are standard Tukey Box Plots, where the bounds of the shaded box represent the first and third quartile; whiskers indicate the minimum and maximum data point within 1.5 times the interquartile range. Mean is denoted with an ‘X.’ Data used to generate the plots have been superimposed on the graph as open circles (n = 12 biologically independent experiments) (*p < 0.05, a→ p < 0.05 comparing trained and untrained students within a given temperature when both restrictive and permissive temperatures are tested, b→ p = ns comparing trained and untrained students within a given temperature when both restrictive and permissive temperatures are tested).

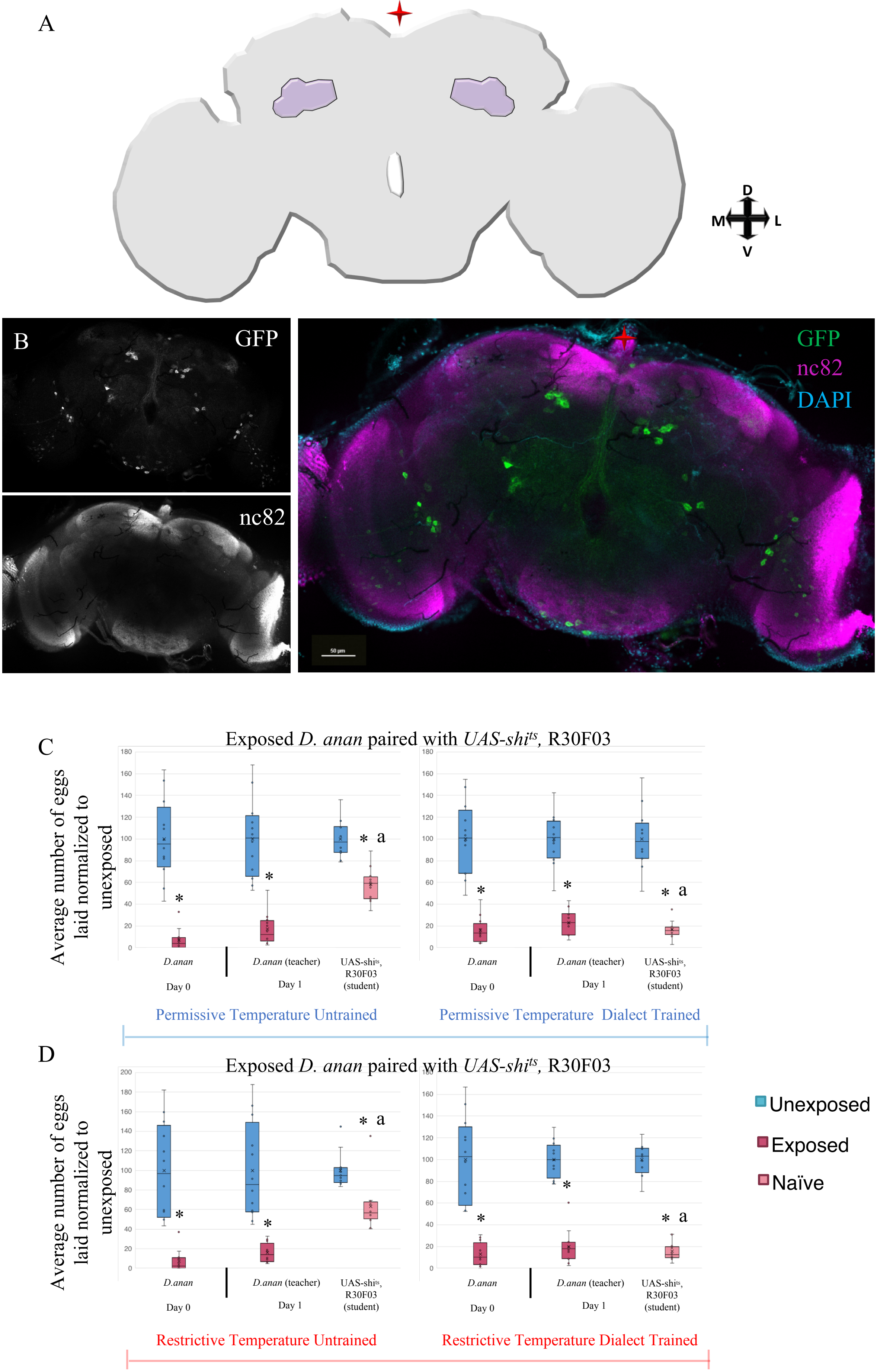

A
D
M
L
V
B
GFP
nc82
GFP
nc82
DAPI
50 µm
C
Exposed D. anan paired with UAS-shits, R30F03
Average number of eggs laid normalized to unexposed
D.anan
Day 0
D.anan (teacher)
Day 1
UAS-shits, R30F03 (student)
* a
Permissive Temperature Untrained
Permissive Temperature Dialect Trained
D
Exposed D. anan paired with UAS-shits, R30F03
Average number of eggs laid normalized to unexposed
Restrictive Temperature Untrained
Restrictive Temperature Dialect Trained
Unexposed
Exposed
Naïve

Supplementary Figure 15. Or47b and Or65a are dispensable for dialect training.

(A) Expression of Or47b in a female fly using the $Or47b^{GFP}$ construct highlighting expression in the antenna. Dotted boxes indicate regions of magnification. Percentage of eggs laid by exposed flies normalized to eggs laid by unexposed flies is shown. $Or47b^{-/-}$ show wild-type trained and untrained behavior (B). (C) Expression of Or47b in a male fly using the $Or47b^{GFP}$ construct highlighting expression in the antenna. $Or49b^{GAL4}$ driving $Or49b^{RNAi}$ shows wild-type untrained behavior and can learn the dialect from *D. ananassae* following training (D). (E) Expression of Or65a in a female fly using the $Or65a^{GFP}$ construct highlighting expression in the antenna. $Or65a^{-/-}$ show wild-type trained and untrained behavior (F). (G) Expression of Or65a in a male fly using the $Or65a^{GFP}$ construct highlighting expression in the antenna. $Or65a^{GAL4}$ driving $Or65a^{RNAi}$ shows wild-type untrained behavior and can learn the dialect from *D. ananassae* following training (H). Blue lines indicate permissive temperature. Trained and untrained states are labeled. Plots are standard Tukey Box Plots, where the bounds of the shaded box represent the first and third quartile; whiskers indicate the minimum and maximum data point within 1.5 times the interquartile range. Mean is denoted with an 'X.' Data used to generate the plots have been superimposed on the graph as open circles (n = 12 biologically independent experiments) (*$p < 0.05$, a→ $p < 0.05$ comparing trained and untrained students within a given temperature, c→ p = ns comparing trained and untrained students in experiments with no restrictive temperature).

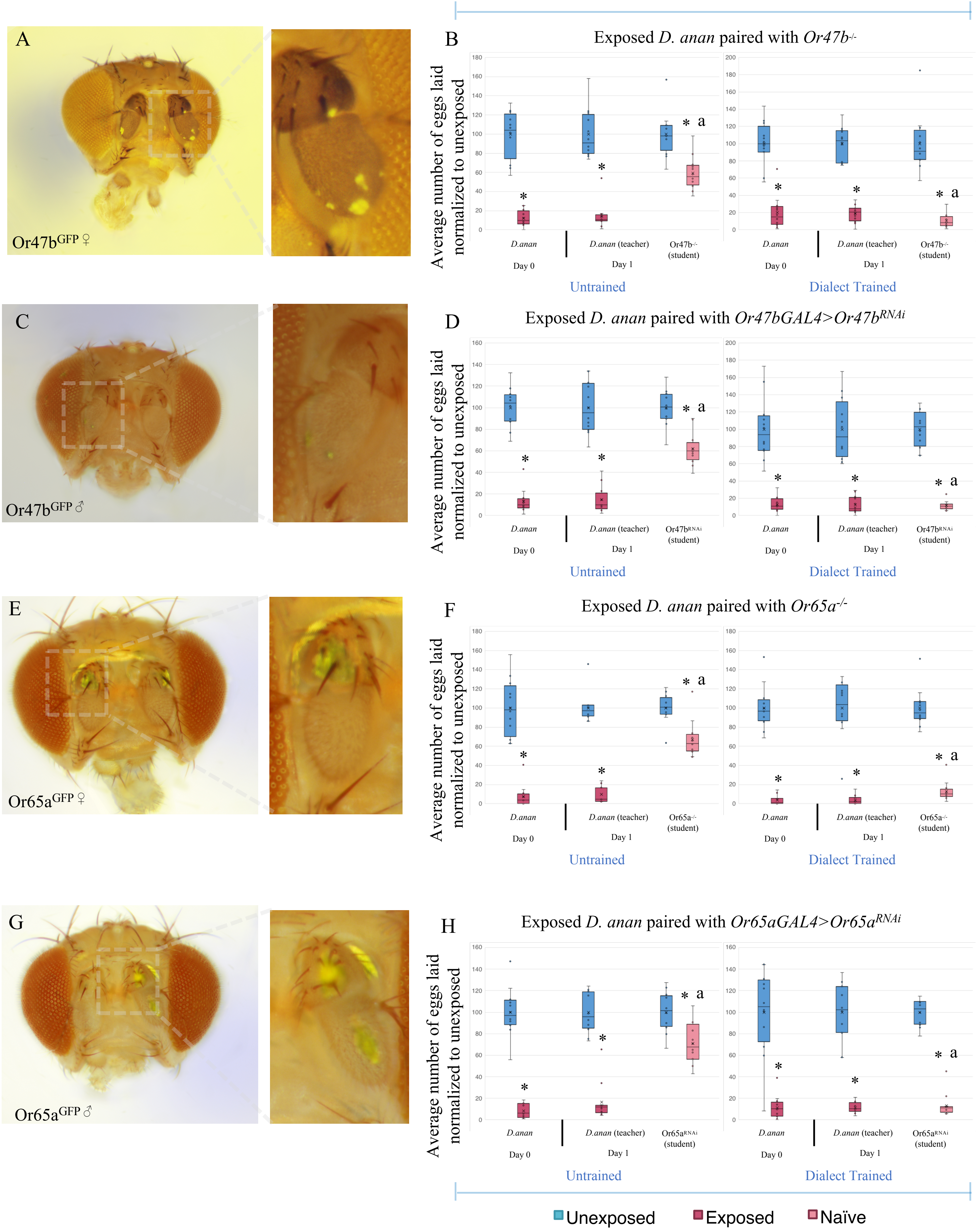

A
Or47b^{GFP} ♀
B
Exposed *D. anan* paired with *Or47b*$^{-/-}$
Average number of eggs laid normalized to unexposed
D.anan
Day 0
D.anan (teacher)
Day 1
Or47b$^{-/-}$ (student)
Untrained
Dialect Trained
C
Or47b^{GFP} ♂
D
Exposed *D. anan* paired with *Or47bGAL4>Or47b*^{RNAi}
Average number of eggs laid normalized to unexposed
D.anan
Day 0
D.anan (teacher)
Day 1
Or47b^{RNAi} (student)
Untrained
Dialect Trained
E
Or65a^{GFP} ♀
F
Exposed *D. anan* paired with *Or65a*$^{-/-}$
Average number of eggs laid normalized to unexposed
D.anan
Day 0
D.anan (teacher)
Day 1
Or65a$^{-/-}$ (student)
Untrained
Dialect Trained
G
Or65a^{GFP} ♂
H
Exposed *D. anan* paired with *Or65aGAL4>Or65a*^{RNAi}
Average number of eggs laid normalized to unexposed
D.anan
Day 0
D.anan (teacher)
Day 1
Or65a^{RNAi} (student)
Untrained
Dialect Trained
Unexposed
Exposed
Naïve

Supplementary Figure 16. Further evidence implicating the role of *Or69a* in dialect training.

(A) Expression of Or69a in male flies using the $Or69a^{GFP}$ construct highlighting expression in the antenna. Dotted boxes indicate regions of magnification. Percentage of eggs laid by exposed flies normalized to eggs laid by unexposed flies is shown. $Or69a^{GAL4}/+$ and $Or69a^{RNAi}/+$ show wild-type trained behavior (B-C). Outcrossed $Or69a^{-/+}$ shows wild-type untrained behavior and dialect training from *D. ananassae* (D). Blue lines indicate permissive temperature. Trained and untrained states are labeled. Plots are standard Tukey Box Plots, where the bounds of the shaded box represent the first and third quartile; whiskers indicate the minimum and maximum data point within 1.5 times the interquartile range. Mean is denoted with an 'X.' Data used to generate the plots have been superimposed on the graph as open circles (n = 12 biologically independent experiments) (*$p < 0.05$, a→ $p < 0.05$ comparing trained and untrained students within a given temperature).

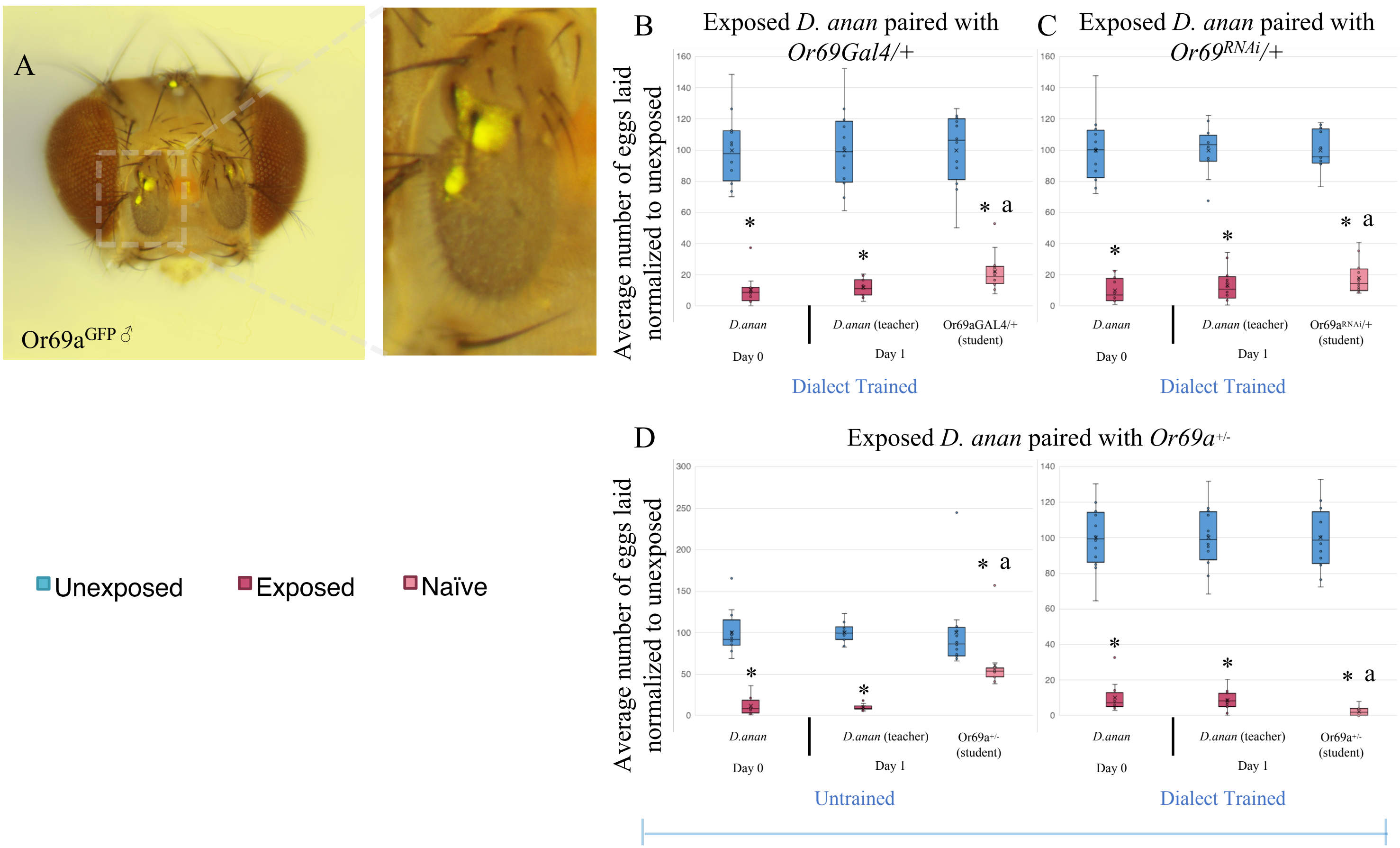

A
Or69aGFP ♂
B
Exposed D. anan paired with Or69Gal4/+
C
Exposed D. anan paired with Or69RNAi/+
Average number of eggs laid normalized to unexposed
D.anan
Day 0
D.anan (teacher)
Day 1
Or69aGAL4/+ (student)
Or69aRNAi/+ (student)
Dialect Trained
D
Exposed D. anan paired with Or69a+/-
Or69a+/- (student)
Untrained
Dialect Trained
Unexposed
Exposed
Naïve

Supplementary Figure 17. Further evidence implicating the role of motion-detecting circuitry in dialect training using UAS Shi^{ts}.

Dialect training is performed at either the permissive (22°C) or restrictive (30°C) temperature, while the wasp exposure and social learning period is performed exclusively at the restrictive temperature. Percentage of eggs laid by exposed flies normalized to eggs laid by unexposed flies is shown. (A) UAS-shi^{ts} crossed to $L1^{GAL4}$ in the untrained state at restrictive and permissive temperatures shows wild-type untrained state. Expression pattern of $L1^{GAL4}$ also shown. (B) UAS-shi^{ts} crossed to $L2^{GAL4}$ in the untrained state at restrictive and permissive temperatures shows wild-type untrained state. Expression pattern of $L2^{GAL4}$ also shown. (C) UAS-shi^{ts} crossed to $L3^{GAL4}$ in the untrained state at restrictive and permissive temperatures shows wild-type untrained state. Expression pattern of $L3^{GAL4}$ also shown. (D) UAS-shi^{ts} crossed to $L4^{0987\text{-}GAL4}$ in the untrained state at restrictive and permissive temperatures shows wild-type untrained state. Expression pattern of $L4^{0987\text{-}GAL4}$ also shown. Plots are standard Tukey Box Plots, where the bounds of the shaded box represent the first and third quartile; whiskers indicate the minimum and maximum data point within 1.5 times the interquartile range. Mean is denoted with an 'X.' Data used to generate the plots have been superimposed on the graph as open circles (n = 12 biologically independent experiments) (*$p < 0.05$, a→ $p < 0.05$ comparing trained and untrained students within a given temperature when both restrictive and permissive temperatures are tested, b→ p = ns comparing trained and untrained students within a given temperature when both restrictive and permissive temperatures are tested. Comparison data is located in Figure 3).

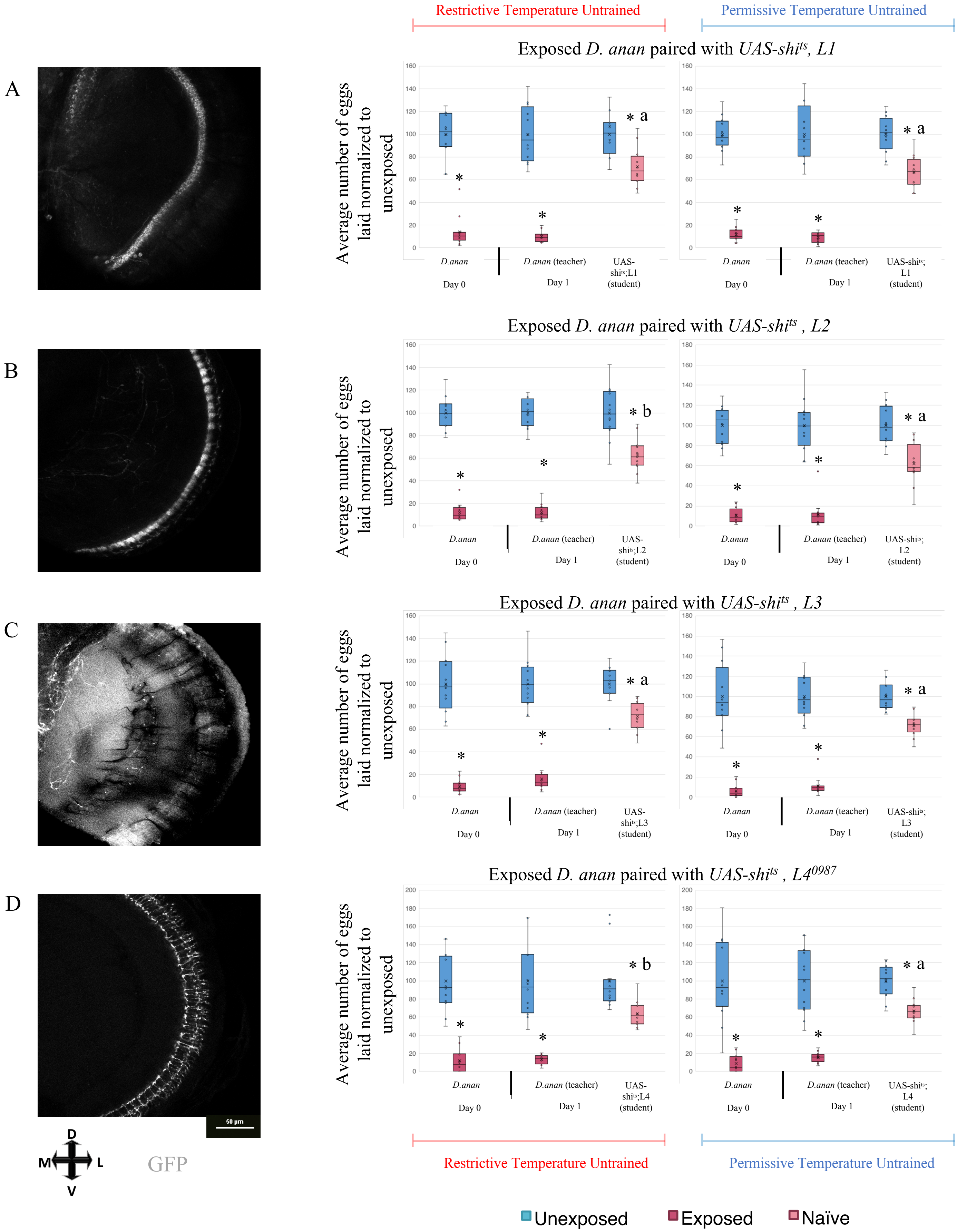

Restrictive Temperature Untrained
Permissive Temperature Untrained
A
Exposed D. anan paired with UAS-shi^ts, L1
B
Exposed D. anan paired with UAS-shi^ts , L2
C
Exposed D. anan paired with UAS-shi^ts , L3
D
Exposed D. anan paired with UAS-shi^ts , L4^0987
Average number of eggs laid normalized to unexposed
D.anan
Day 0
D.anan (teacher)
Day 1
UAS-shi^ts;L1 (student)
UAS-shi^ts;L2 (student)
UAS-shi^ts;L3 (student)
UAS-shi^ts;L4 (student)
* a
* b
50 µm
D
M
L
V
GFP
Restrictive Temperature Untrained
Permissive Temperature Untrained
Unexposed
Exposed
Naïve

Supplementary Figure 18. L4 motion sensing neurons in the optic lobe are required for dialect training.

Dialect learning is performed at either the permissive (22°C) or restrictive (30°C) temperature, while the wasp exposure and social learning period is performed exclusively at the permissive temperature. (A) Confocal image of adult brain where *splitL4*^{GAL4} is driving UAS-CD8-GFP, stained with nc82 (magenta) and DAPI (teal). A magnification is shown of boxed area. UAS-shi^{ts} crossed to *splitL4*^{GAL4} trained by *D. ananassae* at the permissive temperature shows wild-type trained state, but at the restrictive temperature shows defective acquisition in the trained state. (B) UAS-shi^{ts} crossed to split*L4*^{GAL4} in the untrained state at restrictive and permissive temperatures shows wild-type untrained state. Expression pattern of *splitL4*^{GAL4} also shown. Plots are standard Tukey Box Plots, where the bounds of the shaded box represent the first and third quartile; whiskers indicate the minimum and maximum data point within 1.5 times the interquartile range. Mean is denoted with an 'X.' Data used to generate the plots have been superimposed on the graph as open circles (n = 12 biologically independent experiments) (*p < 0.05, a→ p < 0.05 comparing trained and untrained students within a given temperature when both restrictive and permissive temperatures are tested, b→ p = ns comparing trained and untrained students within a given temperature when both restrictive and permissive temperatures are tested).

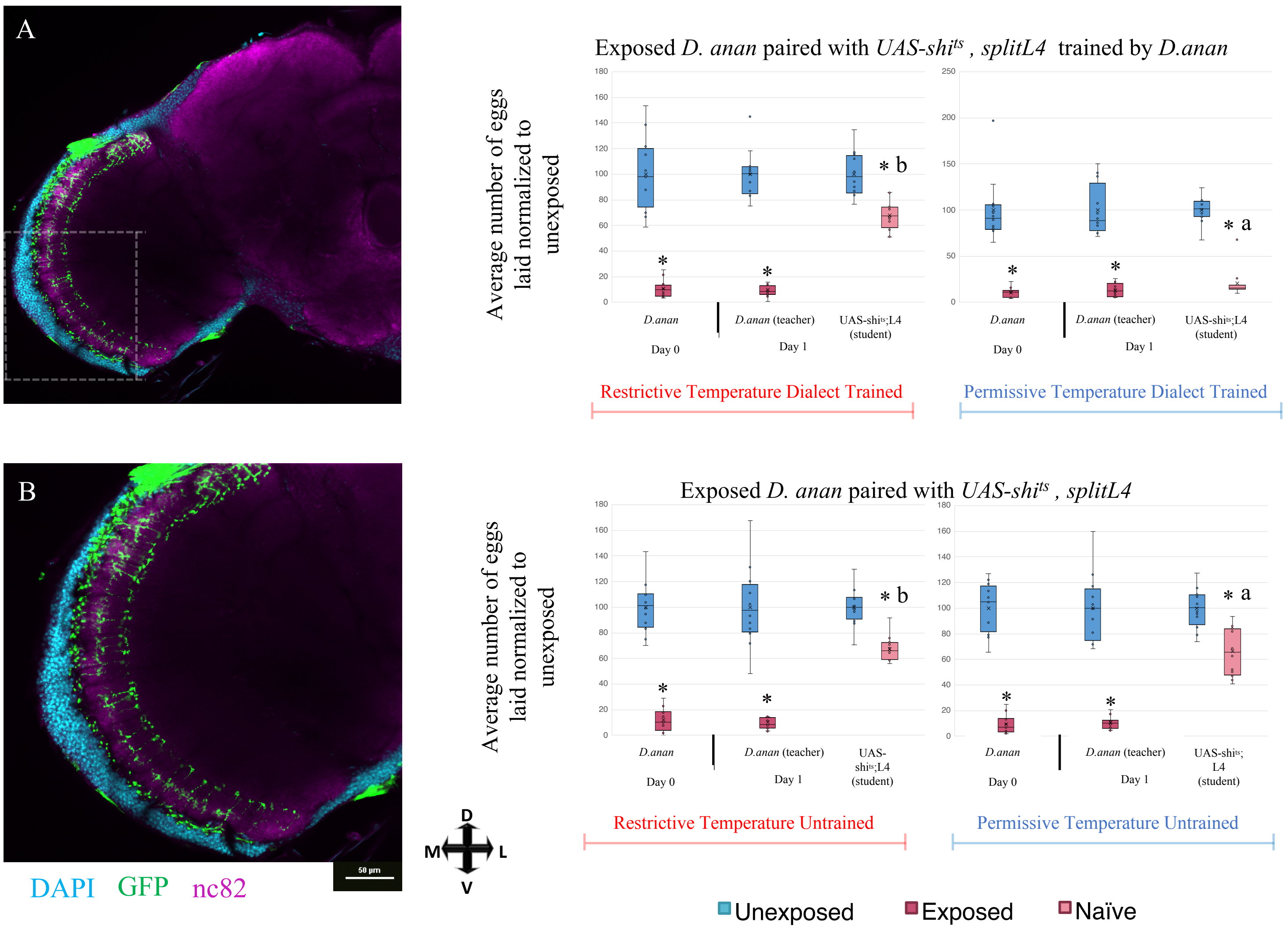

A
B
DAPI
GFP
nc82
50 µm
D
M
L
V
Exposed D. anan paired with UAS-shits , splitL4 trained by D.anan
Average number of eggs laid normalized to unexposed
D.anan
D.anan (teacher)
UAS-shits;L4 (student)
Day 0
Day 1
* b
* a
Restrictive Temperature Dialect Trained
Permissive Temperature Dialect Trained
Exposed D. anan paired with UAS-shits , splitL4
Restrictive Temperature Untrained
Permissive Temperature Untrained
Unexposed
Exposed
Naïve

Supplementary Figure 19. Further evidence indicating that Region 5 of the fan-shaped body is necessary for dialect training.

Dialect learning is performed at either the permissive (22°C) or restrictive (30°C) temperature, while the wasp exposure and social learning period is performed exclusively at the restrictive temperature. Percentage of eggs laid by exposed flies normalized to eggs laid by unexposed flies is shown. (A) Expression and schematic of expression pattern of the R75G12^{GAL4} FSB driver shows pan-FSB marking. (B) UAS-shi^{ts} crossed to R75G12^{GAL4} trained by *D. ananassae* at the permissive and restrictive temperature shows wild-type untrained state. (C) Expression and schematic of expression pattern of the R38E07^{GAL4} FSB driver shows expression in regions 5, 8, and 9. (D) UAS-shi^{ts} crossed to R38E07^{GAL4} trained by *D. ananassae* at the permissive and restrictive temperature shows wild-type untrained state. (E) Expression and schematic of expression pattern of the R89E07^{GAL4} FSB driver shows expression in regions 2, 8, and 9. (F) UAS-shi^{ts} crossed to R89E07^{GAL4} trained by *D. ananassae* at the permissive and restrictive temperature shows wild-type untrained state. (G) Expression and schematic of expression pattern of the R49H02^{GAL4} FSB driver shows expression in regions 1, 4, and 6. (H) UAS-shi^{ts} crossed to R49H02^{GAL4} trained by *D. ananassae* at the permissive and restrictive temperature shows wild-type untrained state. Plots are standard Tukey Box Plots, where the bounds of the shaded box represent the first and third quartile; whiskers indicate the minimum and maximum data point within 1.5 times the interquartile range. Mean is denoted with an 'X.' Data used to generate the plots have been superimposed on the graph as open circles (n = 12 biologically independent experiments) (*$p < 0.05$, a→ $p < 0.05$ comparing trained and untrained students within a given temperature when both restrictive and permissive temperatures are tested, b→ p = ns comparing trained and untrained students within a given temperature when both restrictive and permissive temperatures are tested. Comparison data is located in Figure 4).

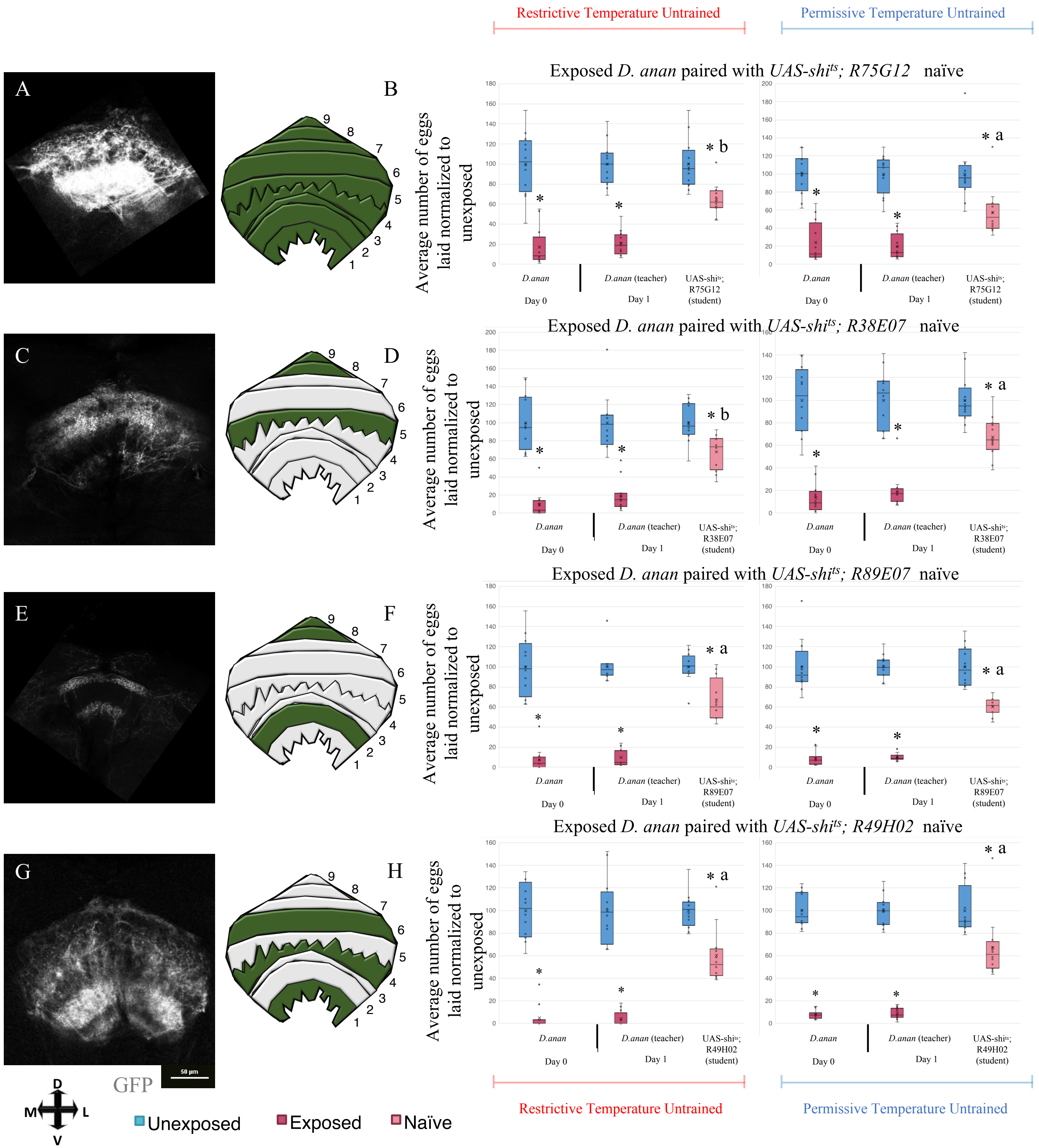
Restrictive Temperature Untrained
Permissive Temperature Untrained
A
B
Exposed D. anan paired with UAS-shits; R75G12 naïve
C
D
Exposed D. anan paired with UAS-shits; R38E07 naïve
E
F
Exposed D. anan paired with UAS-shits; R89E07 naïve
G
H
Exposed D. anan paired with UAS-shits; R49H02 naïve
Average number of eggs laid normalized to unexposed
D.anan
Day 0
D.anan (teacher)
Day 1
UAS-shits; R75G12 (student)
UAS-shits; R38E07 (student)
UAS-shits; R89E07 (student)
UAS-shits; R49H02 (student)
* a
* b
50 µm
GFP
D
M
L
V
Unexposed
Exposed
Naïve

Supplementary Figure 20. Super resolution microscopy of key brain regions in dialect learning.

(A) Z-stack maximum intensity project super resolution image of driver line R38E07 in conjunction with UAS-CD8-GFP. (B) Z-stack maximum intensity projection super resolution image of the antennal lobe stained with nc82. (C) Z-stack maximum intensity projection super resolution image of driver line $L4^{0987}$ in conjunction with UAS-CD8-GFP. (D) Brain regions identified as involved in dialect learning—the optic lobe (blue), mushroom body (orange), antennal lobe (teal), lateral horn (green), fan-shaped body (red), and ellipsoid body (light green). (E) Brain regions identified as being dispensable for dialect learning—the prow (light green), bulb (yellow), and superior clamp (purple).

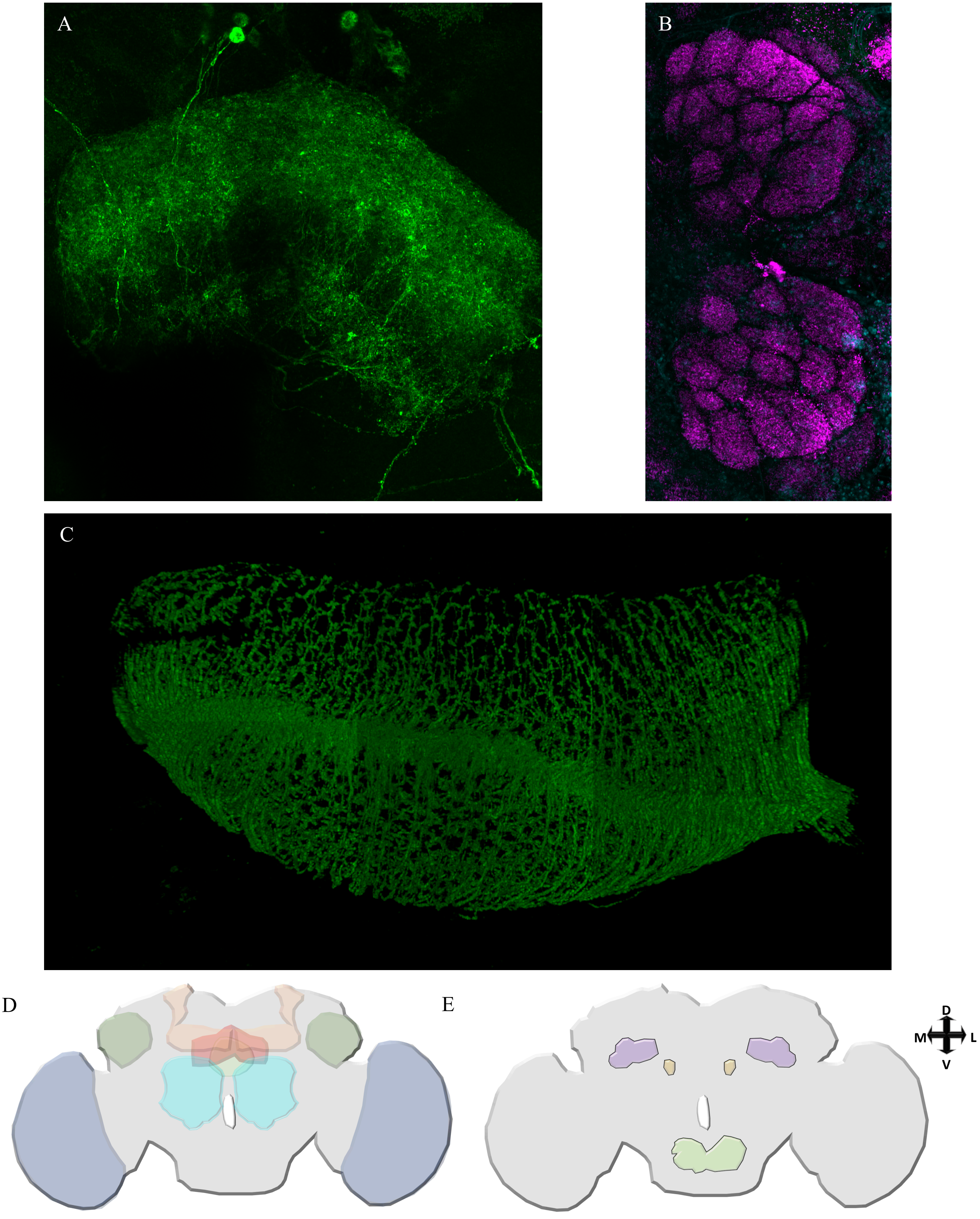
A
B
C
D
E
D
M
L
V

SUPPLEMENTARY TABLES

Supplementary Table 1. Name, genotype, acquisition location, and stock identification number (if applicable) are shown.

Gene/Allele	Genotype	Acquisition location	ID #
CS (WT control)	-	n/a	n/a
D. ananassae	-	Drosophila species stock center	14024-0371.13
L1 GAL4	w[+] (I); L1[c202a]Gal4 / (Cyo) (isoD1)	Marion Silies--European Neuroscience Institute	n/a
L2 GAL4	w[+] (I); L2[21Dhh]-Gal4 / TM6B (isoD1)	Marion Silies--European Neuroscience Institute	n/a
L3 GAL4	w[+] (I); L3[0595]-Gal4 / TM6B	Marion Silies--European Neuroscience Institute	n/a
L4 GAL4	w (I); L4 [0987]-GAL4 / (CyO) (isoD1)	Marion Silies--European Neuroscience Institute	n/a
splitL4 GAL4	w (I); L4 [0987]-GAL4-F32.1-A30-dw (II); 0980-D33.1-A2-dw (III) (isoD1)	Marion Silies--European Neuroscience Institute	n/a
Or69a-GFP	w[*]; P{w[+mC]=Or69aA-Mmus\Cd8a.GFP}3	Bloomington Drosophila Stock Center	52636
Or65a-GFP	w[*]; P{w[+mC]=Or65a-Mmus\Cd8a.GFP}2	Bloomington Drosophila Stock Center	52631
Or47b-GFP	y[1] w[*]; P{w[+mC]=Or47b-CD2.M}3/TM3, Sb[1]	Bloomington Drosophila Stock Center	9915
Or47b-/-	y[1] w[*]; Mi{y[+mDint2]=MIC}Or47b[MI05132]	Bloomington Drosophila Stock Center	38008
Or47b[RNAi]	y[1] v[1]; P{y[+t7.7] v[+t1.8]=TRiP.JF02586}attP2	Bloomington Drosophila Stock Center	27274
Or47b[GAL4]	w[*]; P{w[+mC]=Or47b-GAL4.7.467}15.6	Bloomington Drosophila Stock Center	9984
Or69a-/-	y[1] w[*]; Mi{y[+mDint2]=MIC}Or69a[MI00896]	Bloomington Drosophila Stock Center	32714
Or69a[GAL4]	w[*]; P{w[+mC]=Or69a-GAL4.F}81.3	Bloomington Drosophila Stock Center	9999
Or65a-/-	w[*]; P{w[+mC]=Or65a-GAL4.F}72.5	Bloomington Drosophila Stock Center	9993
Or65a[RNAi]	y[1] v[1]; P{y[+t7.7] v[+t1.8]=TRiP.JF02763}attP2	Bloomington Drosophila Stock Center	27683
Or65a[GAL4]	w[*]; P{w[+mC]=Or65a-GAL4.F}72.5	Bloomington Drosophila Stock Center	9993
Or69a[RNAi]	y[1] sc[*] v[1]; P{y[+t7.7] v[+t1.8]=TRiP.HMC05836}attP40	Bloomington Drosophila Stock Center	64962
optic lobe-GAL4 R10B01	w[1118]; P{y[+t7.7] w[+mC]=GMR10B01-GAL4}attP2	Bloomington Drosophila Stock Center	49234
optic lobe-GAL4 R17F12	w[1118]; P{y[+t7.7] w[+mC]=GMR17F12-GAL4}attP2	Bloomington Drosophila Stock Center	48779
mushroombody-GAL4 R13F02	w[1118]; P{y[+t7.7] w[+mC]=GMR13F02-GAL4}attP2	Bloomington Drosophila Stock Center	48571
mushroombody-GAL4 R16A06	w[1118]; P{y[+t7.7] w[+mC]=GMR16A06-GAL4}attP2	Bloomington Drosophila Stock Center	48709
antenna lobe-GAL4 R21C11	w[1118]; P{y[+t7.7] w[+mC]=GMR21C11-GAL4}attP2	Bloomington Drosophila Stock Center	47898
antenna lobe-GAL4 R14H04	w[1118]; P{y[+t7.7] w[+mC]=GMR19H04-GAL4}attP2	Bloomington Drosophila Stock Center	48865
lateral horn-GAL4 R10G01	w[1118]; P{y[+t7.7] w[+mC]=GMR10G01-GAL4}attP2	Bloomington Drosophila Stock Center	48270
fanshapedbody-GAL4 R75G12	w[1118]; P{y[+t7.7] w[+mC]=GMR75G12-GAL4}attP2	Bloomington Drosophila Stock Center	39906
fanshapedbody-GAL4 R38E07	w[1118]; P{y[+t7.7] w[+mC]=GMR38E07-GAL4}attP2	Bloomington Drosophila Stock Center	50007
fanshapedbody-GAL4 R89E07	w[1118]; P{y[+t7.7] w[+mC]=GMR89E07-GAL4}attP2	Bloomington Drosophila Stock Center	40553
fanshapedbody-GAL4 R49H02	w[1118]; P{y[+t7.7] w[+mC]=GMR58F03-GAL4}attP2	Bloomington Drosophila Stock Center	38712
ellipsoidbody-GAL4 R11F03	w[1118]; P{y[+t7.7] w[+mC]=GMR11F03-GAL4}attP2	Bloomington Drosophila Stock Center	48464
ellipsoidbody-GAL4 R15F02	w[1118]; P{y[+t7.7] w[+mC]=GMR15F02-GAL4}attP2	Bloomington Drosophila Stock Center	48698
bulb-GAL4 R32B03	w[1118]; P{y[+t7.7] w[+mC]=GMR32B03-GAL4}attP2	Bloomington Drosophila Stock Center	49886
prow-GAL4 R25G04	w[1118]; P{y[+t7.7] w[+mC]=GMR25G04-GAL4}attP2	Bloomington Drosophila Stock Center	49136
superiorclamp-GAL4 R30F03	w[1118]; P{y[+t7.7] w[+mC]=GMR30F03-GAL4}attP2	Bloomington Drosophila Stock Center	49640
UAS-shi[ts]	w[*]; P{w[+mC]=UAS-shi[ts1].K}3	Bloomington Drosophila Stock Center	44222
CD8-GFP	UAS-CD8-GFP/Cyo	Mani Ramaswami	n/a
