# Supplementary material for: Neural circuitry of social learning in Drosophila requires multiple inputs to facilitate inter-species communication
Source: Commun Biol. 2019 Aug 13;2:309. doi: 10.1038/s42003-019-0557-5 (PMC6692349; doi:10.1038/s42003-019-0557-5)
Supplement: Supplementary file 2 — Description of Additional Supplementary Files [file 42003_2019_557_MOESM2_ESM.docx]

**Description of additional supplementary items**

**SUPPLEMENTARY DATA FILE LEGEND**

**Supplementary Data 1**. Raw egg counts and p values for figures used in this study. Each tab corresponds to a given panel series. Within tab contains raw egg counts and p-values.

**SUPPLEMENTARY MOVIE LEGENDS**

**Supplementary Movie 1**. Expression pattern of the L4^0987^ line in whole brain.

Super resolution confocal movie of adult Drosophila brain where L4^0987-GAL4^ is driving UAS-CD8-GFP, stained with DAPI (teal) and nc82 (magenta).

**Supplementary Movie 2**. Expression pattern of the L4^0987^ line in optic lobe.

Super resolution confocal movie of adult Drosophila brain where L4^0987-GAL4^ is driving UAS-CD8-GFP, stained with DAPI (teal) and nc82 (magenta).

**Supplementary Movie 3**. Expression pattern of the fan-shaped body driver line R38E07^GAL4^.

Super resolution confocal movie of adult Drosophila brain where R38E07^GAL4^ is driving UAS-CD8-GFP, stained with DAPI (teal).

**Supplementary Movie 4**. Expression pattern of the fan-shaped body driver line R38E07^GAL4^ in a magnified region.

Super resolution confocal movie of adult Drosophila brain where R38E07^GAL4^ is driving UAS-CD8-GFP, stained with DAPI (teal).
