# Supplementary material for: Neural circuitry of social learning in Drosophila requires multiple inputs to facilitate inter-species communication
Source: Commun Biol. 2019 Aug 13;2:309. doi: 10.1038/s42003-019-0557-5 (PMC6692349; doi:10.1038/s42003-019-0557-5)
Supplement: Supplementary file 3 — Reporting Summary [file 42003_2019_557_MOESM3_ESM.pdf]

nature research

Corresponding author(s): Balint Z Kacsoh and Giovanni Bosco

Last updated by author(s): Jul 15, 2019

Reporting Summary

Nature Research wishes to improve the reproducibility of the work that we publish. This form provides structure for consistency and transparency in reporting. For further information on Nature Research policies, see Authors & Referees and the Editorial Policy Checklist.

Statistics

For all statistical analyses, confirm that the following items are present in the figure legend, table legend, main text, or Methods section.

n/a	Confirmed	
☐	☒	The exact sample size (n) for each experimental group/condition, given as a discrete number and unit of measurement
☐	☒	A statement on whether measurements were taken from distinct samples or whether the same sample was measured repeatedly
☐	☒	The statistical test(s) used AND whether they are one- or two-sided<br>*Only common tests should be described solely by name; describe more complex techniques in the Methods section.*
☐	☒	A description of all covariates tested
☐	☒	A description of any assumptions or corrections, such as tests of normality and adjustment for multiple comparisons
☐	☒	A full description of the statistical parameters including central tendency (e.g. means) or other basic estimates (e.g. regression coefficient) AND variation (e.g. standard deviation) or associated estimates of uncertainty (e.g. confidence intervals)
☒	☐	For null hypothesis testing, the test statistic (e.g. F, t, r) with confidence intervals, effect sizes, degrees of freedom and P value noted<br>*Give P values as exact values whenever suitable.*
☒	☐	For Bayesian analysis, information on the choice of priors and Markov chain Monte Carlo settings
☒	☐	For hierarchical and complex designs, identification of the appropriate level for tests and full reporting of outcomes
☒	☐	Estimates of effect sizes (e.g. Cohen's d, Pearson's r), indicating how they were calculated

Our web collection on statistics for biologists contains articles on many of the points above.

Software and code

Policy information about availability of computer code

Data collection	*Provide a description of all commercial, open source and custom code used to collect the data in this study, specifying the version used OR state that no software was used.*
Data analysis	Microsoft excel was used for statistical analysis.

For manuscripts utilizing custom algorithms or software that are central to the research but not yet described in published literature, software must be made available to editors/reviewers. We strongly encourage code deposition in a community repository (e.g. GitHub). See the Nature Research guidelines for submitting code & software for further information.

Data

Policy information about availability of data

All manuscripts must include a data availability statement. This statement should provide the following information, where applicable:

- Accession codes, unique identifiers, or web links for publicly available datasets
- A list of figures that have associated raw data
- A description of any restrictions on data availability

Data availability
All raw data presented is available in this paper and its supplement.

Field-specific reporting

Please select the one below that is the best fit for your research. If you are not sure, read the appropriate sections before making your selection.

☒ Life sciences ☐ Behavioural & social sciences ☐ Ecological, evolutionary & environmental sciences

For a reference copy of the document with all sections, see nature.com/documents/nr-reporting-summary-flat.pdf

Life sciences study design

All studies must disclose on these points even when the disclosure is negative.

Sample size	An n of 12 samples per experiment were utilized. No statistical predetermination was performed. We selected this N based on previous publications.
Data exclusions	No data were excluded from analysis.
Replication	Experiments were replicated utilizing multiple controls and conditions. Blinding and various experimenters were further employed to ensure reproducibility.
Randomization	Samples, in particular GAL4 driver lines and mutants, were randomized and coded before testing such that experimenters were unaware of genotype until the end of the experiments.
Blinding	Experimenters were blinded to treatment condition and genotype prior to quantification by an individual not involved in the quantification process to ensure total blinding. Experimenter coding did not count and vice versa. At least 3 experimenters were involved in coding multiple facets of experiment at all times. Genotype was coded by numerical code throughout study such that experimenter setting up crosses or assays did not know what genotype was being tested. Lines were only decoded following completion of experiments to ensure total unbiased approach.

Reporting for specific materials, systems and methods

We require information from authors about some types of materials, experimental systems and methods used in many studies. Here, indicate whether each material, system or method listed is relevant to your study. If you are not sure if a list item applies to your research, read the appropriate section before selecting a response.

Materials & experimental systems

n/a	Involved in the study	
☐	☒	Antibodies
☒	☐	Eukaryotic cell lines
☒	☐	Palaeontology
☐	☒	Animals and other organisms
☒	☐	Human research participants
☒	☐	Clinical data

Methods

n/a	Involved in the study	
☒	☐	ChIP-seq
☒	☐	Flow cytometry
☒	☐	MRI-based neuroimaging

Antibodies

Antibodies used	nc82 antibody (Developmental Studies at Hybridoma Bank, University of Iowa, Registry ID AB 2314866
Validation	Initial Publication<br><br>Bruchpilot, a protein with homology to ELKS/CAST, is required for structural integrity and function of synaptic active zones in Drosophila.<br>Buchner E.<br>Neuron 49.6 (2006 Mar 16): 833-44.

Animals and other organisms

Policy information about studies involving animals; ARRIVE guidelines recommended for reporting animal research

Laboratory animals	The D. melanogaster strain Canton-S (CS) was used as the wild-type strain and used for outcrosses. The Drosophila species D. ananassae and D. virilis were acquired from the Drosophila Species Stock Center (DSSC) at the University of California, San Diego, stock numbers 14024-0371.13 and 15010-1051.87, respectively. L1, L2, L3, L40987, and splitL4GAL4 lines were kindly provided by Marion Silies (European Science Institute, Germany). The CD8-GFP line was kindly provided by Mani Ramaswami

(Trinity College, Dublin Ireland). All stocks used in experiments are listed in Table S1 with stock numbers shown (when applicable). Flies were aged 3-5 prior to experimentation.

Wild animals	This study did not utilize observation of wild animals.
Field-collected samples	We utilized the generalist Figitid larval endoparasitoid Leptopilina heterotoma (strain Lh14), that is known to infect a wide array of Drosophilids. L. heterotoma strain Lh14 originated from a single female collected in Winters, California in 2002. To propagate wasp stocks, we used adult D. virilis, which were placed in batches of 40 females and 15 males per vial (Genesse catalog number 32-116). The strain we used has been maintained on D. virilis since 2013, though was originally maintained on D. melanogaster. Adult flies are allowed to lay eggs in these standard Drosophila vials that contain 5 mL standard Drosophila media supplemented with live yeast (approximately 25 granules) for 4-6 days. Flies were then replaced by adult wasps—15 female and 6 male wasps—for infections. Infection timing gives the wasps access to the L2 stage of D. virilis larvae. Vials that contain wasps are supplemented with approximately 500 µL of a 50% honey/water solution that is applied to the inside of the cotton vial plugs. The honey used was organic, raw and unfiltered. Wasps aged 3-7 days post eclosion were used for all infections and experiments. Wasps were never reused for experiments, nor were they used for stock propagation if used for experiments.
Ethics oversight	*Identify the organization(s) that approved or provided guidance on the study protocol, OR state that no ethical approval or guidance was required and explain why not.*

Note that full information on the approval of the study protocol must also be provided in the manuscript.
